# Stable, Environmental Specific and Novel QTL Identification as Well as Genetic Dissection of Fatty Acid Metabolism in *Brassica napus*

**DOI:** 10.3389/fpls.2018.01018

**Published:** 2018-07-17

**Authors:** Binghao Bao, Hongbo Chao, Hao Wang, Weiguo Zhao, Lina Zhang, Nadia Raboanatahiry, Xiaodong Wang, Baoshan Wang, Haibo Jia, Maoteng Li

**Affiliations:** ^1^College of Life Science and Technology, Huazhong University of Science and Technology, Wuhan, China; ^2^Hybrid Rapeseed Research Center of Shaanxi Province, Shaanxi Rapeseed Branch of National Centre for Oil Crops Genetic Improvement, Yangling, China; ^3^Provincial Key Laboratory of Agrobiology, Key Laboratory of Cotton and Rapeseed, Ministry of Agriculture, Institute of Industrial Crops, Jiangsu Academy of Agricultural Sciences, Nanjing, China; ^4^College of Life Science, Shandong Normal University, Jinan, China; ^5^Hubei Collaborative Innovation Center for the Characteristic Resources Exploitation of Dabie Mountains, Huanggang Normal University, Huanggang, China

**Keywords:** *Brassica napus*, fatty acid, QTL mapping, acyl lipid metabolism, genetic dissection

## Abstract

Fatty acid (FA) composition is the typical quantitative trait in oil seed crops, of which study is not only closely related to oil content, but is also more critical for the quality improvement of seed oil. The double haploid (DH) population named KN with a high density SNP linkage map was applied for quantitative trait loci (QTL) analysis of FA composition in this study. A total of 406 identified QTL were detected for eight FA components with an average confidence interval (CI) of 2.92 cM, the explained phenotypic variation (PV) value ranged from 1.49 to 45.05%. Totally, 204 consensus and 91 unique QTL were further obtained via meta-analysis method for the purpose of detecting multiple environment expressed and pleiotropic QTL, respectively. Of which, 74 stable expressed and 22 environmental specific QTL were also revealed, respectively. In order to make clear the genetic mechanism of FA metabolism at individual QTL level, conditional QTL analysis was also conducted and more than two thousand conditional QTL which could not be detected under the unconditional mapping were detected, which indicated the complex interrelationship of the QTL controlling FA content in rapeseed. Through comparative genomic analysis and homologous gene annotation, 61 candidates related to acyl lipid metabolism were identified underlying the CI of FA QTL. To further visualize the genetic mechanism of FA metabolism, an intuitive and meticulous network about acyl lipid metabolism was constructed and some closely related candidates were positioned. This study provided a more accurate localization for stable and pleiotropic QTL, and a deeper dissection of the molecular regulatory mechanism of FA metabolism in rapeseed.

## Introduction

Rapeseed (*Brassica napus* L., genome AACC, 2n = 38) is the second largest oil crop in the world, which can provide affluent edible oil for diet and valuable source for industrial biodiesel (Kimber and Mcgregor, 1995). High oil cultivars undoubtedly could increase the oil production, and high quality property is more important for the seed oil in rapeseed. Although possessing the high level of unsaturated FA, such as oleic acid (about 60% proportion), which is a tangible advantage, some disadvantages such as the high level of erucic acid is also one of the limitations for the utilization of rapeseed oil (Zhao et al., 2008). Thus, revealing the genetic mechanism of FA biosynthesis, increasing the production and improving the quality of seed oil, are the main purpose of breeding program in *B. napus*.

FA in *B. napus* mainly includes palmitic acid (C16:0), stearic acid (C18:0), oleic acid (C18:1), linoleic acid (C18:2), linolenic acid (C18:3), eicosenoic acid (C20:1), erocic acid (C22:1), and this FA profile has been frequently influenced by the breeding environment (Si et al., 2003). QTL mapping has been applied in detecting the QTL of enormous and important agronomic traits among various crops, such as rice (McCouch and Doerge, 1995), wheat (Sourdille et al., 2000), and *B. napus* (Burns et al., 2003). Despite several studies have been performed for the QTL analysis of FA in rapeseed, the deficiencies of lower resolution and credibility, poor precision of the detected QTL still exist due to few years and sites of field trial, low marker density, and smaller size of the mapping populations were used (Burns et al., 2003; Hu et al., 2006; Zhao et al., 2008; Smooker et al., 2011; Yan et al., 2011; Wang et al., 2015), and finally obtained results were hardly applied to practical breeding program due to lower credibility and large CI. With the development of sequencing technologies and the advances in analytical methods (Chalhoub et al., 2014; Wang et al., 2015), these defections can be made up by using higher marker density in mapping population. More importantly, the effort for identification of environmental stable and specific QTL to meet the actual demand of breeding program in *B. napus* is also profound and the field trials with multiple breeding sites, as well as multiyear can facilitate goal achievement.

Seed oil of *B. napus* commonly contains seven FA components (Velasco and Becker, 1998). Previous QTL studies showed that except for C22:1 which was controlled by two major QTL, other traits were genetically controlled by enormous QTL and meanwhile displayed complex interaction with the environment (Burns et al., 2003; Qiu et al., 2006; Zhao et al., 2008; Smooker et al., 2011; Yan et al., 2011). Burns et al. (2003) identified 27 QTL that were distributed across nine linkage groups of the genome and involved seven FA compositions in *B. napus*, 19 out of the 27 QTL were also related to the trait of oil content. 38 and 34 QTL were revealed by Zhao et al. (2008) and Smooker et al. (2011), respectively, and both of them were related to the seven main FA compositions and were located in sixteen linkage groups of *B. napus* except for A4, A5 and A10. Yan et al. (2011) used a mapping population with 183 lines and 40 QTL controlling six FA compositions were detected, of which 21 QTL were located in N8 and N13 linkage groups. By increasing the marker density and population size, Wang et al. (2015) utilized a population with 202 lines and a middle density linkage map containing 932 markers to identify 72 QTL, which were associated to 10 FA compositions and distributed in 17 linkage groups of *B. napus* except for C2 and C4. Despite lots of FA QTL analyses were carried out, more elaborate effort associating with the reliable and practical QTL detection is still indispensable for the QTL fine mapping analysis of FA in *B. napus*.

As the classical and reliable analytical method, conventional linkage analysis for detecting QTL of various corps has been utilized for decades despite its requirement of artificial population construction and multiyear field trials. In recent years, Genome-wide associated analysis (GWAS) was also widely used for QTL revealing associated with diverse traits in rapeseed (Gacek et al., 2016; Qu et al., 2017; Wan et al., 2017). On account of the disadvantages of the poor ability in false positive controlling and in rare allele detection (El-Soda et al., 2015), the traditional linkage analysis is still an invaluable method for QTL detection especially accompanied by other advanced analytical methods.

*B. napus* was originated from natural hybridization between *B. rapa* and *B. oleracea* about 7500 years ago, the outcome of this event is that the allotetraploid *B. napus* possessed the larger size and more complex architecture of genome and many genes with multiple replicas (Gacek et al., 2016). The biological process of acyl-lipid metabolism in plants were complex and hierarchical, more than 120 different reactions and 600 genes involved in this process were revealed in *Arabidopsis* (Li-Beisson et al., 2013). Although many efforts for exploring QTL and genes related to acyl-lipid metabolism in *Arabidopsis* were implemented (Li-Beisson et al., 2013), few knowledge are clear in this process in *B. napus*. The close genetic relationship between *Arabidopsis* and *B. napus* as well as the release of their genome sequence (Parkin et al., 2003; Chalhoub et al., 2014) enable us to carry out gene function prediction of acyl-lipid metabolism based on QTL fine-mapping in *B. napus*. *De novo* biosynthesis pathway of acyl-lipid in rapeseed is a complicated process that contains many significant procedures, such as the synthesis of long chain FA from acetyl-CoA, lipid trafficking, desaturation and elongation reaction of the synthesized FA and finally the production of TAG along with its storage in seeds (Ohlrogge and Browse, 1995; Li-Beisson et al., 2013). Generally, these metabolic processes involved diverse genes and were genetically controlled by various regulators; furthermore, the crosstalk among these genes could also be observed regularly. Actually, acyl-lipid metabolism related genes were generally regulated in a coordinated manner during the seed development in plants (Baud and Lepiniec, 2009).

Besides studies of complex metabolic regulatory network at the whole level, and the genetic mechanism investigation at individual trait level of FA composition in rapeseed have been seldom implemented so far. In addition, despite the phenomenon of high correlation of the PV for different FA compositions could be attributed to the QTL co-localization among them (Burns et al., 2003; Hu et al., 2006; Zhao et al., 2008; Smooker et al., 2011; Yan et al., 2011; Wang et al., 2015), but it insufficiently provides detailed genetic explanation for this phenomenon, as two basic facts for some loci with multiple effect and/or some genes closely linked and located in the same locus contributed largely to the correlation of PV for different traits are difficult to distinguish by common QTL analysis method (Shi et al., 2009). So, in order to solve this confusion, the method of conditional QTL mapping was used to explore the genetic relationships between two closely correlated traits at individual QTL level (Wen and Zhu, 2005). This method enables us to study the phenotypic variation of one FA trait under the condition of excluding the influence from another related trait. Hence, for the aim to get a more profound understanding of the genetic mechanism for FA content accumulation, it is necessary for us to carry out QTL analysis at the level of individual trait in rapeseed.

In recent years, many key genes involved in FA biosynthesis were positioned and identified in plants. Several FA biosynthetic initiation and elongation enzymes were well characterized. For example, the *A. thaliana* gene fatty acid biosynthesis 1 (*FAB1*), which responses for the elongation of C16:0-ACP to C18:0-ACP, the key step for FA synthesis process (Chapman and Burke, 2012), was positioned in two linkage groups of A2 and C1 of *B. napus* by genome-wide association mapping method (Qu et al., 2017). Another *A. thaliana* gene long-chain acyl-CoA synthetase 9 (*LACS9*), which catalyzes the formation of acyl-CoA that involved in *Arabidopsis* seed oil biosynthesis (Zhao et al., 2010), was also detected and positioned in A2 linkage group in this study. Two important FA desaturation enzymes *FAD2* and *FAD3* were mapped in the linkage groups of A1, A3, A5, C1, and C5 (Scheffler et al., 1997; Schierholt et al., 2000; Yang et al., 2012; Wang et al., 2015), and linkage groups of A3, A4, A5, A8, A10, C3, C4, and C5 (Hu et al., 2006; Smooker et al., 2011; Wang et al., 2015), respectively. The functional study revealed that these two genes produced C18:1 and C18:3 using plastidial FAs through a desaturation modification, respectively (Okuley et al., 1994; Yang et al., 2012). Basnet et al. (2016) reported another two *FAD* family genes *BrFAD5* and *BrFAD7* that could interact with one same family gene *BrFAD2* to affect the content of oleic and linoleic in *B. rapa*. A multifunctional gene *FAE1* (FA elongation 1), which was responsible for the formation of C22:1 and TAG from FA (James et al., 1995), was positioned in five linkage groups of A1-A4 and A8 in *B. napus* (Wang et al., 2015). Lately, Shi et al. (2017) reported that depressing the expression of *FAD2* and *FAE1* led to the increased content of oleic acid while significantly decreased the content of erucic acid and slightly reduced the oil content of seed in *B. napus*. Although pathways and many genes involved acyl-lipid metabolism have been well-characterized, the genetic regulatory mechanisms of FA biosynthesis network are still largely unknown. Due to the complicated genome structure of rapeseed, solving of this subject is challenging as usual.

The PV of FA trait in oil corps was not only directly controlled by a large amount of genes, but also was affected by the digenic interaction among them, such as epistatic effect (Jourdren et al., 1996). Previous studies showed that the epistatic interaction effect between the alleles was a basic genetic component for the quantitative trait and always played a vital role in the quantitative trait for the genetic variation and evolution of crops (Li et al., 2001). Lü et al. (2011) reported the epistatic association mapping in homozygous crop cultivars. Singh et al. (2013) studied the genetic factors involved in stem rust resistance and explored the epistatic interaction among them in spring wheat. Li et al. (2012) also conducted the QTL and epistatic analyses related to the seed yield trait in rapeseed. Though many epistatic effect studies were conducted in crops, few of them were about this effect in rapeseed up to now. In view of the importance of the formation on the genetic basis of QTL, the study of epistatic effect should be taken into consideration in QTL analysis process.

In this study, the unconditional and conditional QTL mapping analysis of FA traits were performed based on a high density linkage map, and a plenty of potential candidate genes involving acyl-lipid metabolism were revealed. The aims of the present study were: (1) revealing the stable and environmental specific FA related QTL and candidate genes of rapeseed with higher precision and credibility; (2) providing deeper understanding of the genetic basis of FA metabolism during the seed development in rapeseed; (3) giving better guidance for breeding high quality rapeseed varieties.

## Materials and methods

### Plant material, field planting, concentration determination, and correlation analysis for FA compositions

The segregating double haploid (DH) population with 348 lines used in this study was derived from the cross of “KenC-8” × “N53-2” and was initially constructed by Wang et al. (2015), here it was named KN DH population. The DH lines and the parents were planted in three independent macroenvironments associated with three Provinces in China and 14 microenvironments were involved. Briefly, the breeding sites of Dali (DL) and Yangling (YL) associate with Shanxi Province and seven microenvironments of 08DL, 09DL, 10DL, 11DL, 12DL, 13DL, and 14YL (7 years from 2008 to 2014, planting in the areas) were tested and all of them belong to winter type environment. Gansu (GS) associates with Gansu Province and two microenvironments of 10GS and 11GS were tested and two of them belong to spring environment. Wuhan (WH) and Huanggang (HG) associate with Hubei Province and five microenvironmets of 11WH, 12WH, 13WH, 14WH, and 11HG were tested and all of them belong to semi-winter environment. Wuhan and Huanggang were the experiment bases of Huazhong University of Science and Technology of Hubei Province, and Dali, Yangling and Gansu were the experiment bases of Hybrid Rapeseed Research Center of Shanxi Province. The field trails were complemented as the same as reported by (Wang et al., 2015) that all lines were planted in a randomized complete-block designed with three replicates and no specific permissions were required. A total of seven traits were studied and the total amount of all saturated FA compositions was called one trait of FAS. The content for each trait was measured by near-infrared reflectance spectroscopy method (Mika et al., 2003) and take the average of 3 replicates. Pearson correlation analysis among traits was performed by using SPSS 19.0 software (SPSS Inc., Chicago, IL, USA).

### QTL mapping and epistasis analysis

A high density SNP-based linkage map that included more than 3000 markers with an average genetic distance of 0.96 cM was constructed for KN DH population (Chao et al., 2017). The map was used for QTL mapping and epistasis analysis for FAs together with phenotype data in the current study.

QTL and epistasis analyses were performed by using WinQTLCart_2.5 and QTLNetwork_2.0 software as (Chao et al., 2017) descripted, respectively. QTLs detected via unconditional analysis and integrated QTL after two rounds of meta-analysis were directly called identified, consensus and unique QTL and then were named with initial prefix of “uq,” “ucq,” and “uuq,” respectively. The naming patterns of identified QTL and consensus QTL were similar as consisting of the prefix plus the trait and followed by the linkage group. For example, “uqLA-A8-1” and “ucqLA-A8-1” represented the first identified and consensus QTL for trait LA and located in A8 linkage, respectively. The naming pattern of unique QTL was similar to that of identified and consensus QTL which just not contained the trait. For example, the unique QTL of uuqC3-2 represented the second unique QTL of C3 linkage group. The identified QTLs with overlapping CI for the same trait and repeatedly detected in different microenvironments were integrated into consensus QTL through meta-analysis by using BioMercator 2.1 software with default parameters (Arcade et al., 2004). Consensus QTLs detected in one microenvironment and with PV>20% or detected in more than one microenvironment with PV>10% were considered as the major QTLs. Consensus QTLs controlling the same traits and with the overlapping CI were further integrated into unique QTLs and the ones that had no overlapping CI with others were also considered as unique QTLs.

The conditional phenotypic values of y(T1|T2) were predicted by using QGAStation1.0 software (Zhao et al., 2006), where T1|T2 indicating the meaning of trait 1 conditioned on trait 2. For example, y(PA|SA) was the conditional phenotypic value of PA conditioned on SA, it means that the obtained phenotypic value for PA without the influence from the trait of SA. The QTL detected from the conditional analysis were called conditional identified and consensus QTL and then were named with the initial prefix of “cq” and “ccq,” respectively. The naming pattern for the conditional QTL was identical to that of unconditional analysis.

### Identification of the potential candidates related to acyl lipid metabolism, and genetic interaction analysis of the candidate genes

On account of the collinearity relationship of *B. napus* and its reference genome together with the massive different alleles obtained from the re-sequencing for the parents of “KenC-8” and “N53-2” (Chao et al., 2017), the alleles which existed within the unique region and had SNP or InDel variation in intron, exon or within 1 kb up and down stream between the two parents were regarded as candidate genes.

The visualized interaction network was constructed by String software (http://string-db.org/) and exhibited by Cytoscape V-3.5.0 software (Shannon et al., 2003). Nodes represent the potential candidates and edges represent the interaction of them. Node size represented “Degree” and edge size represented “Combined-score,” the color for nodes and edges represented the “Betweenness centrality” and “Edge Betweenness,” respectively. All the value for these four parameters was calculated by Network Analyzer that included in Cytoscape V-3.5.0 software.

### Quantitative PCR (qPCR) analysis for five acyl lipid metabolism related potential candidates

The silicles for qPCR analysis were obtained at 15, 30, and 45 days after flowing (DAF) within two lines of materials and with the high C18:1 content (mean 55.22% for the all measured microenvironments) and the low C18:1 content (mean 18.87% for the all measured microenvironments), respectively. For each different developmental stage, three biological replicate samples from were used for expression analysis of each candidate. Seed were stripped from the silicles of the three plants for the next total Procedure of RNA extraction experiment followed the manufacturer's protocol of RNAprep Pure Plant Kit (TIANGEN, DP441, China). cDNA was synthesized from 2 mg total RNA using HiScript II Q RT SuperMix for qPCR (+gDNA wiper) (Vazyme, R223-01, China), RNA expression level analysis was performed using AceQ qPCR SYBR Green Master Mix (Vazyme, Q141-02/03, China) using StepOnePlus™ Real-Time PCR System (ThermoFisher Scientific, USA). For each reaction, three technical replicates were validated. p values were calculated through Student's *t*-test by using SPSS 19.0 software (SPSS Inc., Chicago, IL, USA). The primer pairs of the five candidate genes and reference gene Actin are listed in (Table S9).

### Construction of potential regulatory pathway of FA metabolism in *B. napus*

The Kyoto Encyclopedia of Genes and Genomes (KEGG) database (http://www.kegg.jp/) was applied to construct the regulatory pathways in which the FA metabolism related candidates revealed in this study were involved. The potential regulatory pathways of FAs metabolism were inferred based on that of *Arabidopsis*. Six major processes of plastidial FA synthesis and elongation, TAG synthesis and degradation, β-oxidation, phospholipid and choline metabolism and lipoic aicd metabolism were integrated and included in this pathway.

## Results

### Phenotypic variation and correlation analysis of FA compositions in *B. napus*

**Figure 1:**
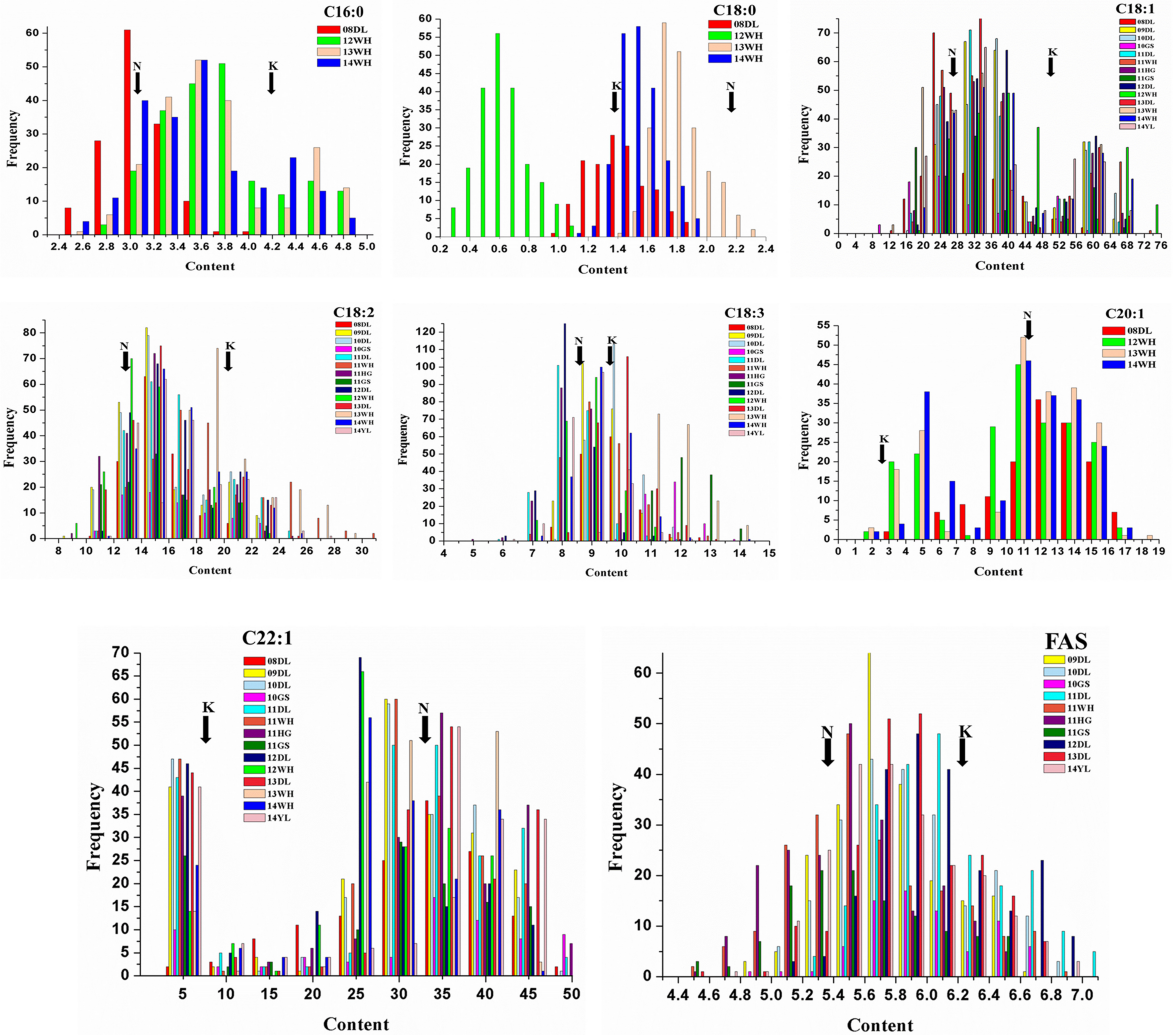
Distribution of FA concentrations of KN DH population in multiple environments. The unit of x-axis represents the percentage of each FA composition in the sum of all FAs. The unit of y-axis represents the number of lines. K represents the male parent of “KenC-8” and N represents the female parent of “N53-2” of the KN-DH population.

The mean concentration for the single FA composition varied a lot with the range of 1.3–34.05% and showed transgressive segregation performance compared with that of the parents (Figure 1). The three most abundant compositions, C18:1, C22:1 and C18:2, possessed the mean content of 34.05, 26.48, and 15.53%, and the variation coefficient was 9.07, 8.57, and 9.08%, respectively (Table 1). The three monounsaturated FAs (MUFAs) C18:1, C20:1, and C22:1 displayed bi-modal distribution pattern, which indicated that these compositions might be controlled by a few major genes with a relatively large effect. The frequency distribution of the remaining five FA compositions displayed normal or near-normal distribution, which implied that these compositions were typical for the quantitative composition and were controlled by various loci with small genetic effect (Figure 1). Different compositions in the oilseed generally have the overt positive or negative correlation with each other. The Pearson correlation analysis showed that the two MUFAs C20:1 and C22:1 had significant negative correlation with the other six compositions, with the mean correlation coefficient of −0.696 and −0.803, respectively. However, a high positive correlation between these two compositions was observed with correlation coefficient of 0.857 (*P* < 0.01). Besides C20:1 and C22:1, the remaining six compositions have totally positive correlation with each other (Table S1). For example, the correlation coefficient between C16:0 and C18:2 upped to a high value of 0.917, this suggested a close relationship in the two compositions during the acyl lipid metabolism process.

**Table 1:** The mean and range of content for each fatty aicd composition in KN DH population with 14 microenvironments field trial.

	**C16:0**	**C18:0**	**C18:1**	**C18:2**	**C18:3**	**C20:1**	**C22:1**	**FAS**
N53-2 ± SD	3.12 ± 0.24	2.18 ± 2.10	26.41 ± 10.70	12.86 ± 3.07	8.28 ± 0.73	11.35 ± 4.21	34.04 ± 12.46	5.38 ± 0.28
Ken-C8 ± SD	4.21 ± 0.33	1.41 ± 0.57	51.58 ± 12.58	19.93 ± 2.70	9.65 ± 1.00	4.12 ± 1.11	6.63 ± 11.45	6.27 ± 0.44
Min ± SD	2.46 ± 0.16	0.9 ± 0.49	13.27 ± 3.68	10.3 ± 1.91	7.07 ± 1.56	1.44 ± 0.86	0.54 ± 0.66	5 ± 0.49
Max ± SD	4.68 ± 0.52	1.77 ± 0.52	63.23 ± 4.44	23.67 ± 2.59	12.27 ± 1.22	16.1 ± 0.56	46.58 ± 4.13	6.14 ± 1.11
Mean ± SD	3.42 ± 0.31	1.3 ± 0.50	34.05 ± 3.09	15.53 ± 1.41	9.18 ± 1.29	10.16 ± 0.82	26.48 ± 2.27	6 ± 0.39
CV(%)	9.06	38.46	9.07	9.08	14.05	8.07	8.57	6.5

*SD, Standard Deviation; CV, Coefficient of Variance; “KenC-8” × “N53-2” represents the male and female parent line, respectively*.

### Stable and environmental specific QTL analysis for FA composition

**Figure 2:**
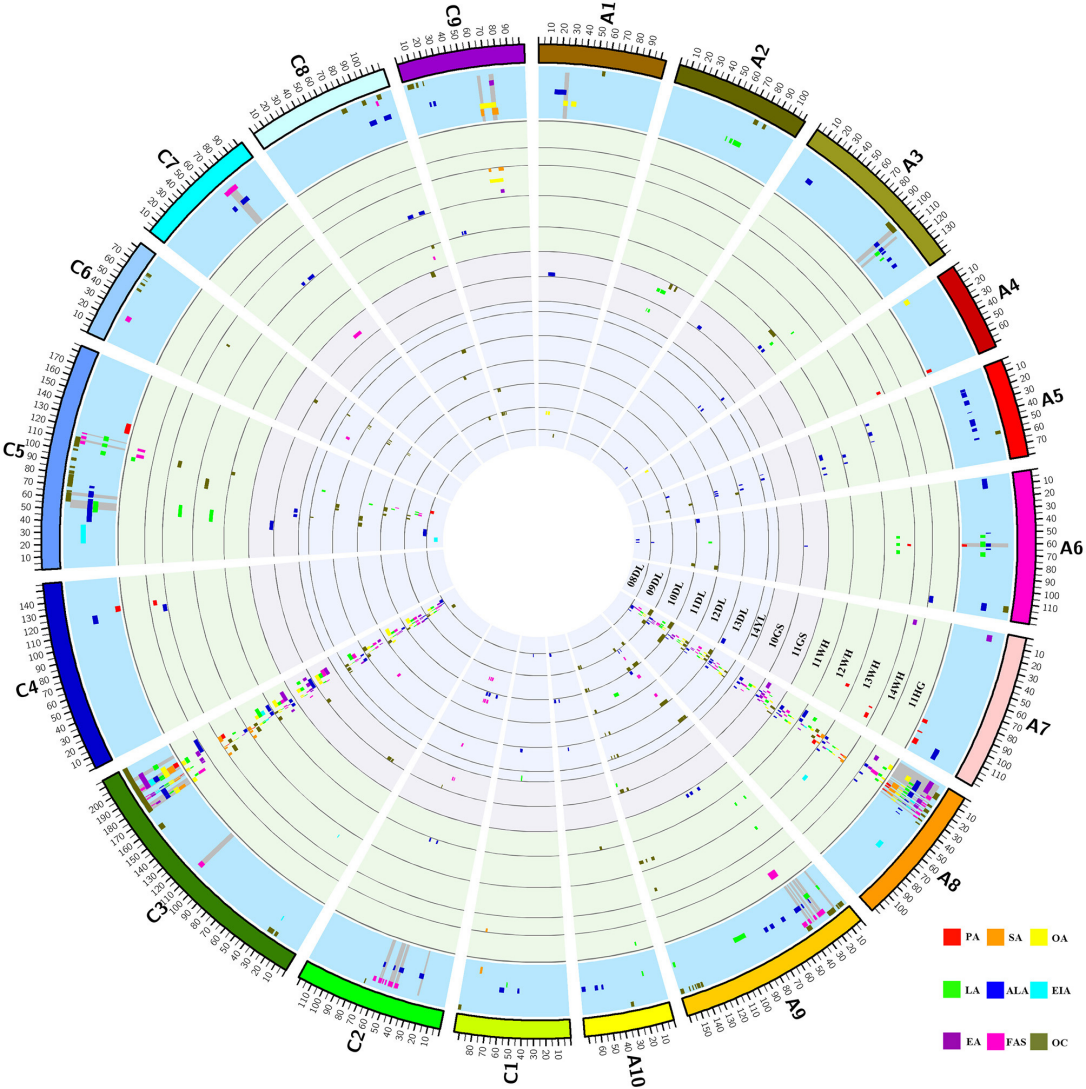
Distribution of QTL for each FA and oil content in each linkage group. The entire graph consists of 16 circles. From the inside to outside, the first 14 circles represent the 14 breeding microenvironments, it contains seven winter type of 08DL to 14YL, two spring type of 10GS and 11GS and five semi-winter type of 11WH to 11 HG, identified QTL for the nine traits located in each circle and indicated by different colors. The fifteenth circle with blue background represents the region includes consensus QTL for each trait and the gray spokes highlight the region of unique QTL. The outermost circle represents the 19 linkage groups of *B. napus* and they also distinguished by different colors. The abbreviation of PA, SA, OA, LA, ALA, EIA, EA, FAS, and OC represents the traits of C16:0, C18:0, C18:1, C18:2, C18:3, C20:1, C22:1, FAS, and oil content, respectively.

A total of 406 identified QTL were detected, it was revealed that 211 and 195 QTL were distributed in the A and C genome, respectively. The number of identified QTL for the single component varied from 9 (C20:1) to 111 (C18:3) and these QTL explain the maximum PV of 45.05% for uqEA-12DL8-1. Further analysis showed that 67.73% of these identified QTL were located on A8 and C3 linkage groups, two of which respectively contains 137 and 138 QTL and they formed the distribution clusters (Figure 2; Figures S1, S2; Table S2).

The 406 identified QTL were integrated into 204 consensus QTL through meta-analysis for the purpose of detecting multiple environment expressed QTL (Table 2). The number of consensus QTL for single composition ranged from 9 (C20:1) to 63 (C18:3) and the single QTL explaining PV varied from 1.49 to 40.53%. A total of 81 major QTL were identified, the largest PV value upped to 45.05% explained by ucqEA-A8-6. For the single composition, there were 5, 5, 14, 13, 11, 7, 14, and 12 major QTL for C16:0, C18:0, C18:1, C18:2, C18:3, C20:0, C22:1, and FAS were obtained, respectively. Majority of these major QTL (94, accounting for 46.08%) were located on A8 and C3 linkage groups (Figure S1). For the entire 204 consensus QTL, 52 QTL were detected in at least two macroenvironments and 74 QTL were detected in at least two microenvironments. For example, ucqOA-A8-3, ucqOA-C3-4, ucqLA-A8-4, ucqLA-C3-6, ucqALA-A8-3, ucqEA-A8-9, ucqEA-C3-5, ucqFAS-A8-4 and ucqFAS-C3-2 were observed simultaneously in three macroenvironments. Especially, two QTL of ucqOA-C3-7 and ucqALA-C3-4 were detected in eight microenvironments of the three macroenvironments. These QTL, also represent the major ones, which were constantly detected in at least two years for one or more macroenvironments could be defined as “environmental stable QTL” and it's also indicated that they were slightly affected by the breeding environments. More importantly, a total of 22 environmental specific QTL were revealed, all of which were expressed only in specific macroenvironments. In addition, a few novel QTL, such as ucqPA-C5, were also discovered based on the utilization of the high-resolution DH population here (Table 2).

**Table 2:** The consensus QTL revealed in KN DH population for fatty acid compositions.

**Traits**	**C-QTL^a^**	**Chr^b^**	**Peak**	**CI**	**LOD**	**Additive**	**PV^c^**	**Environments**
C16:0	*ucqPA-A4*	A04	69.61	68.6–71.4	3.56	0.08	1.75	13WH
	*ucqPA-A6*	A06	62.21	60.9–62.9	5.11	0.09	2.55	13WH
	*ucqPA-A7-1*	A07	97.51	94.4–97.8	8.99	−0.21	5.69	12WH
	*ucqPA-A7-2*	A07	106.91	106.6–107.6	5.73	−0.10	2.87	13WH
	*ucqPA-A7-3*	A07	114.51	114.2–119.5	5.33	−0.09	2.73	13WH
	***ucqPA-A8-1***	A08	25.90	25.1–26.8	32.31	−0.26	26.85	12WH
	***ucqPA-A8-2***	A08	28.10	28–28.8	46.58	−0.34	37.23	13WH
	***ucqPA-A8-3***	A08	32.30	31.96–32.65	24.31–34.56	−0.31–0.23	21.90–32.05	**12WH/13WH**
	***ucqPA-C3-1***	C03	184.41	184.33–184.48	35.16–37.11	−0.30–0.28	26.50–30.32	**12WH/13WH**
	***ucqPA-C3-2***	C03	194.71	192.7–197.1	21.77	−0.27	21.55	13WH
	*ucqPA-C4*	C04	124.31	123.9–129	3.78	0.14	6.67	14WH
	*ucqPA-C5*	C05	129.41	125.8–135.1	4.84	−0.09	11.95	08DL
C18:0	***ucqSA-A8-1***	A08	23.61	21.6–25.9	18.92	−0.08	22.58	12WH
	***ucqSA-A8-2***	A08	27.71	27.6–29.7	21.70	−0.09	24.96	13WH
	***ucqSA-A8-3***	A08	32.30	31.59–33.02	13.49–13.97	−0.07	16.91–17.52	**12WH/13WH**
	*ucqSA-C1*	C01	78.81	78.5–80.2	5.46	−0.08	10.21	14WH
	*ucqSA-C3-1*	C03	174.01	173.6–176	11.49	−0.06	12.95	13WH
	*ucqSA-C3-2*	C03	176.01	173.6–176.4	11.25	−0.07	15.24	12WH
	***ucqSA-C3-3***	C03	182.91	182.35–183.46	17.41–19.92	−0.09–0.08	18.57–24.48	**12WH/13WH**
	***ucqSA-C3-4***	C03	184.41	184.16–184.65	19.24–20.21	−0.09–0.08	20.11–24.64	**12WH/13WH**
	*ucqSA-C3-5*	C03	192.71	190.9–196.8	14.71	−0.07	16.34	13WH
	*ucqSA-C9-1*	C09	63.81	62.2–64.7	6.95	0.04	6.34	13WH
	*ucqSA-C9-2*	C09	74.81	72.8–78.2	5.65	0.04	5.74	13WH
C18:1	*ucqOA-A1-1*	A01	24.91	22.8–26.7	5.33	−1.94	2.56	09DL
	*ucqOA-A1-2*	A01	34.11	30–34.5	6.25	−2.04	2.87	09DL
	*ucqOA-A4*	A04	5.51	1.9–6.3	4.78	−1.77	2.15	09DL
	***ucqOA-A8-1***	A08	11.61	9.1–13.6	18.32	−6.01	20.21	11HG
	***ucqOA-A8-2***	A08	19.66	19.14–20.17	9.93–30.70	−6.67–6.26	22.13–26.46	11DL/12DL/10GS
	***ucqOA-A8-3***	A08	24.11	23.82–24.39	23.47–47.97	−8.81–6.77	26.05–36.21	09DL/10DL/12WH/11HG/11GS
	***ucqOA-A8-4***	A08	25.90	25.72–26.09	37.26–47.72	−9.46–6.82	28.21–35.61	11DL/12DL/13DL/11WH/13WH
	***ucqOA-A8-5***	A08	27.71	27.6–28.4	25.82	−6.57	24.10	11HG
	***ucqOA-A8-6***	A08	28.81	28.49–29.12	35.44–41.11	−7.39–7.07	32.59–35.39	**09DL/10DL**
	***ucqOA-A8-7***	A08	32.90	32.67–33.14	22.43–29.84	−7.84–5.76	19.55–24.83	11DL/12DL/13DL/ 11WH/13WH
	***ucqOA-A8-8***	A08	35.11	34.7–35.6	16.72	−7.65	26.54	11GS
	*ucqOA-C3-1*	C03	174.01	172.72–175.29	3.54–6.51	−3.22–3.19	2.75–3.59	11DL/11HG
	*ucqOA-C3-2*	C03	176.41	176–177.8	4.18	−2.69	1.88	09DL
	***ucqOA-C3-3***	C03	182.80	182.64–182.97	31.17–42.37	−9.33–7.79	33.49–36.97	10DL/11WH/12WH
	***ucqOA-C3-4***	C03	183.43	183.28–183.58	19.39–43.07	−8.02–7.51	28.65–35.62	11DL/11HG/11GS
	***ucqOA-C3-5***	C03	184.41	184.36–184.45	9.44–50.65	−10.02–6.74	20.99–38.84	12DL/13DL/12WH/13WH/10GS
	***ucqOA-C3-6***	C03	185.27	185.07–185.46	17.68–45.25	−9.05–6.59	26.86–36.83	09DL/10DL/11DL/11WH/11HG/11GS
	***ucqOA-C3-7***	C03	191.95	191.0–192.9	12.62–39.37	−9.06–5.01	6.33–34.92	09DL/10DL/11DL/12DL/11WH/12WH/11HG/11GS
	***ucqOA-C3-8***	C03	194.71	189.9–199.8	7.98	−6.96	22.39	10GS
	*ucqOA-C9-1*	C09	67.41	62–70.8	3.53	1.92	1.52	13WH
	*ucqOA-C9-2*	C09	74.81	70.8–76.6	3.98	2.29	2.14	13WH
C18:2	*ucqLA-A2-1*	A02	59.11	58.9–59.5	4.73	1.03	7.07	10GS
	*ucqLA-A2-2*	A02	63.91	62.92–64.89	5.22–6.38	0.93–1.14	5.42–7.69	**10GS/11GS**
	*ucqLA-A2-3*	A02	69.11	66.5–73.5	4.87	0.78	4.26	11GS
	*ucqLA-A3-1*	A03	109.21	108–111.1	3.44	−0.62	2.15	11WH
	*ucqLA-A3-2*	A03	115.81	115.3–116.5	5.58	−0.53	3.31	12WH
	*ucqLA-A6-1*	A06	53.91	52.3–54.7	4.26	0.48	2.26	13WH
	*ucqLA-A6-2*	A06	59.81	58.3–62.1	3.48	0.42	1.65	11DL
	*ucqLA-A6-3*	A06	62.21	60–62.9	5.54	0.55	2.91	13WH
	*ucqLA-A6-4*	A06	70.21	68–71.8	3.99	0.46	2.12	13WH
	***ucqLA-A8-1***	A08	13.93	13.12–14.73	20.07–26.40	−1.62–1.34	20.38–25.97	10DL/12WH/11HG
	***ucqLA-A8-2***	A08	17.61	17.3–17.8	13.69	−1.75	23.78	11GS
	***ucqLA-A8-3***	A08	19.91	19.3–21.1	38.80	−1.82	34.10	10DL
	***ucqLA-A8-4***	A08	23.41	22.75–24.07	10.76–46.04	−1.84–1.56	28.21–35.36	09DL/12WH/11HG/10GS
	***ucqLA-A8-5***	A08	25.98	25.69–26.26	17.10–44.80	−2.15–1.74	17.63–34.39	10DL/12DL/13DL/11WH/13WH/11GS
	***ucqLA-A8-6***	A08	28.81	28.58–29.03	17.17–43.08	−2.03–1.73	29.24–38.20	10DL/11DL/11HG/10GS
	***ucqLA-A8-7***	A08	32.34	32.16–32.52	13.29–32.25	−1.89–1.48	14.63–25.82	09DL/11DL/12DL/13DL/11WH/13WH/11GS
	*ucqLA-A9-1*	A09	10.61	10.1–11.6	4.50	0.49	2.75	12WH
	*ucqLA-A9-2*	A09	21.91	21.6–23.9	4.47	0.52	2.36	13WH
	*ucqLA-A9-3*	A09	25.41	23.9–25.7	5.87	0.57	3.53	12WH
	*ucqLA-A9-4*	A09	90.91	89.5–100.7	3.78	−0.46	1.93	11DL
	*ucqLA-A9-5*	A09	159.01	158.2–160	5.12	−1.03	7.67	10GS
	*ucqLA-A10*	A10	15.61	15.4–16.3	4.01	1.04	3.05	11HG
	*ucqLA-C1*	C01	54.71	54.2–55.1	4.32	−1.31	7.79	14YL
	***ucqLA-C3-1***	C03	176.4	175.95–176.86	7.02–32.10	−1.69–0.92	3.25–27.74	09DL/10DL/11HG
	***ucqLA-C3-2***	C03	182.84	182.66–183.02	8.67–34.15	−1.77–1.44	16.23–30.74	12WH/10GS/11GS
	***ucqLA-C3-3***	C03	184.41	184.36–184.45	40.13–51.29	−2.44–1.73	31.08–39.02	11DL/12DL/13DL/11WH/12WH/13WH
	***ucqLA-C3-4***	C03	185.21	184.95–185.46	27.26–39.72	−1.79–1.70	26.88–31.41	09DL/10DL/11HG
	***ucqLA-C3-5***	C03	190.91	190.3–191.51	23.24–33.73	−1.90–1.47	23.57–30.74	12DL/13DL/12WH/13WH
	***ucqLA-C3-6***	C03	192.83	191.92–193.75	6.16–29.79	−2.36–1.00	3.50–28.27	09DL/10DL/11DL/11WH/11HG/10GS/11GS
	*ucqLA-C3-7*	C03	203.21	201.2–205.2	11.00	−1.56	19.03	11GS
	*ucqLA-C5-1*	C05	54.11	49.25–58.97	3.69–3.93	0.52–0.56	3.07–3.09	**12WH/13WH**
	*ucqLA-C5-2*	C05	104.01	101.5–105	3.44	−0.51	2.61	11HG
	*ucqLA-C5-3*	C05	110.21	108.9–111.5	4.04	−0.44	1.99	10DL
	*ucqLA-C5-4*	C05	111.01	110.1–111.91	4.24–4.88	−0.48–0.46	2.18–2.20	**09DL/11DL**
	*ucqLA-C5-5*	C05	115.21	114.4–116.9	4.03	−0.44	1.78	13DL
	*ucqLA-C5-6*	C05	117.21	116.9–118.6	5.08	−0.47	2.29	09DL
C18:3	*ucqALA-A1*	A01	22.41	14.5–24.7	6.19	0.31	11.02	11GS
	*ucqALA-A3-1*	A03	18.91	17.4–22.8	3.59	0.21	5.33	11GS
	*ucqALA-A3-2*	A03	104.11	101–104.8	7.04	−0.33	8.60	11WH
	*ucqALA-A3-3*	A03	109.41	107.4–110.6	6.89	−0.32	8.42	11WH
	*ucqALA-A3-4*	A03	112.41	111.59–113.22	4.18–6.26	−0.22–0.16	4.39–6.94	**09DL/13DL**
	*ucqALA-A3-5*	A03	120.11	119–121.4	4.56	−0.19	5.13	13DL
	*ucqALA-A3-6*	A03	127.71	126.1–129.2	4.40	−0.24	7.74	14YL
	*ucqALA-A5-1*	A05	12.91	12.5–14.5	3.76	−0.23	4.50	13WH
	*ucqALA-A5-2*	A05	18.61	15.2–19.4	4.30	−0.17	5.14	10DL
	*ucqALA-A5-3*	A05	23.91	22.7–26.8	7.29	−0.32	8.42	13WH
	*ucqALA-A5-4*	A05	33.81	33.65–33.98	4.12–5.74	−0.29–0.24	4.55–7.31	14YL/11WH/13WH
	*ucqALA-A5-5*	A05	40.84	40.3–46.2	4.34–5.70	−0.30–0.18	4.74–8.00	12DL/13DL/14YL/12WH
	*ucqALA-A5-6*	A05	45.91	44–46.2	5.15	−0.21	6.74	12DL
	*ucqALA-A5-7*	A05	49.31	48.6–50.9	5.75	−0.22	7.42	12DL
	*ucqALA-A5-8*	A05	50.50	49.94–51.07	4.09–5.58	−0.24–0.19	4.43–6.56	13DL/11WH/12WH
	*ucqALA-A5-9*	A05	57.81	57.4–59	3.88	−0.23	4.21	11WH
	*ucqALA-A6-1*	A06	4.31	2–10.8	3.98	0.17	4.09	11DL
	***ucqALA-A6-2***	A06	50.62	50.37–50.86	4.94–4.52	0.18–0.29	5.73–10.37	**08DL/09DL**
	*ucqALA-A6-3*	A06	60.01	59.8–61.9	5.20	0.32	11.82	08DL
	*ucqALA-A6-4*	A06	62.01	60–62.6	4.58	0.17	5.42	09DL
	*ucqALA-A6-5*	A06	65.21	64.6–65.6	4.45	0.25	7.69	11GS
	*ucqALA-A6-6*	A06	117.81	113.5–120.1	3.55	0.20	4.98	11HG
	*ucqALA-A7*	A07	118.11	114.5–124.2	3.93	0.19	4.92	13DL
	*ucqALA-A8-1*	A08	11.61	8.1–17.6	10.29	−0.32	14.43	12WH
	*ucqALA-A8-2*	A08	19.91	19.9–21.1	6.08	−0.30	11.35	11GS
	***ucqALA-A8-3***	A08	21.76	20.77–22.75	4.35–12.56	−0.34–0.23	7.34–18.57	11WH/12HG/10GS
	***ucqALA-A8-4***	A08	23.61	22.88–24.33	8.08–12.04	−0.37–0.25	10.00–15.09	09DL/10DL/13DL/11WH/13WH
	***ucqALA-A8-5***	A08	28.81	28.36–29.25	6.91–13.81	−0.42–0.26	12.28–22.69	09DL/13DL/11WH/13WH/10GS/11GS
	***ucqALA-A8-6***	A08	32.31	32.17–32.44	5.44–14.60	−0.42–0.26	9.09–19.04	09DL/10DL/11DL/12DL/13DL/11WH/11HG
	***ucqALA-A8-7***	A08	38.28	37.85–38.72	5.48–9.47	−0.36–0.27	8.52–18.47	10DL/13DL/11WH/13WH/10GS
	***ucqALA-A8-8***	A08	42.01	41.74–42.21	3.96–7.65	−0.26–0.22	6.71–10.04	09DL/11DL/12DL/11HG
	*ucqALA-A9-1*	A09	24.31	23.9–24.8	4.02	−0.17	4.84	10DL
	*ucqALA-A9-2*	A09	31.81	31.3–32.5	5.17	−0.19	6.14	10DL
	*ucqALA-A9-3*	A09	35.61	34.5–40.1	6.79	−0.23	7.84	11DL
	*ucqALA-A9-4*	A09	51.91	51.3–54.4	3.57	−0.24	4.88	11WH
	*ucqALA-A9-5*	A09	63.61	62.9–66.1	6.30	−0.32	8.37	11WH
	*ucqALA-A9-6*	A09	69.61	69.2–72.1	3.63	−0.25	4.98	11WH
	*ucqALA-A10-1*	A10	53.81	52.9–54.4	5.92	−0.23	6.53	13DL
	*ucqALA-A10-2*	A10	59.01	57.9–60.4	4.87	−0.20	5.59	11DL
	*ucqALA-A10-3*	A10	70.60	69.04–72.15	5.92–6.97	−0.23–0.22	6.93–9.2	**09DL/11DL**
	*ucqALA-C1-1*	C01	44.31	43.3–44.8	3.84	0.17	4.07	09DL
	*ucqALA-C1-2*	C01	57.91	57.1–60.9	3.51	0.16	3.51	13DL
	*ucqALA-C2-1*	C02	24.11	23.6–27.4	4.80	0.19	4.90	11DL
	*ucqALA-C2-2*	C02	45.81	42.4–46.2	3.67	0.18	3.79	11DL
	*ucqALA-C2-3*	C02	50.84	50.23–51.45	3.58–4.83	0.18–0.21	4.74–5.57	11DL/12WH
	*ucqALA-C2-4*	C02	59.61	59.2–60.9	4.05	0.20	4.74	12WH
	*ucqALA-C3-1*	C03	174.01	173.6–176.4	4.09	−0.18	5.19	10DL
	*ucqALA-C3-2*	C03	178.06	176.85–179.28	4.15–5.20	−0.29–0.19	5.13–9.68	13DL/11GS
	*ucqALA-C3-3*	C03	182.91	179.2–183.2	7.13	−0.36	9.08	11WH
	***ucqALA-C3-4***	C03	185.42	185.11–185.74	4.31–10.41	−0.40–0.17	4.96–12.54	09DL/10DL/11DL/13DL/11WH/12WH/13WH/11GS
	***ucqALA-C3-5***	C03	193.26	191.33–196.2	3.55–6.18	−0.32–0.16	4.18–11.45	09DL/12WH/11HG/11GS
	*ucqALA-C4*	C04	120.51	116.3–123.5	3.41	0.23	6.11	14WH
	*ucqALA-C5-1*	C05	47.01	40.4–53.9	5.62	0.32	9.95	11GS
	***ucqALA-C5-2***	C05	56.10	51.61–60.59	5.06–8.02	0.25–0.28	10.21–14.41	**09DL/12DL**
	*ucqALA-C5-3*	C05	63.41	62.3–65.3	9.84	0.26	12.00	09DL
	*ucqALA-C5-4*	C05	64.91	63.2–67.8	5.49	0.32	17.24	10GS
	***ucqALA-C5-5***	C05	71.48	70.61–72.34	5.85–7.75	0.23–0.33	9.64–18.20	09DL/10GS
	*ucqALA-C7-1*	C07	64.11	61.9–65.3	3.41	0.22	3.99	13WH
	*ucqALA-C7-2*	C07	72.61	71.6–79.2	5.77	0.28	6.57	13WH
	*ucqALA-C8-1*	C08	95.61	91.3–97.3	3.52	−0.23	3.94	13WH
	*ucqALA-C8-2*	C08	105.91	104.8–111	3.68	−0.24	4.12	13WH
	*ucqALA-C9-1*	C09	19.01	17.8–19.2	5.88	−0.23	6.84	12WH
	*ucqALA-C9-2*	C09	21.91	21–23.2	5.42	−0.22	6.35	12WH
C20:1	***ucqEIA-A8-1***	A08	25.91	25.7–26.9	37.24	2.20	30.58	12WH
	***ucqEIA-A8-2***	A08	32.31	32.1–32.9	30.76	1.98	24.33	13WH
	*ucqEIA-A8-3*	A08	74.81	70.7–76.6	3.60	−0.52	1.72	13WH
	***ucqEIA-C3-1***	C03	25.91	25.6–26.3	51.10	2.41	35.53	13WH
	***ucqEIA-C3-2***	C03	183.21	182.9–183.5	40.15	2.39	34.37	12WH
	***ucqEIA-C3-3***	C03	184.41	184.3–184.5	42.69	2.42	35.72	12WH
	***ucqEIA-C3-4***	C03	192.71	190.9–196.2	25.69	2.09	25.53	12WH
	*ucqEIA-C5*	C05	24.01	21.4–37.6	3.63	0.87	8.94	08DL
	***ucqEIA-C9***	C09	184.41	184.3–184.5	54.99	2.61	40.53	13WH
C22:1	*ucqEA-A7*	A07	6.91	3.6–8.9	3.90	3.67	7.05	14WH
	*ucqEA-A8-1*	A08	6.01	2.6–11.2	5.83	4.57	6.23	11GS
	***ucqEA-A8-2***	A08	11.61	9–13.6	19.45	6.97	23.78	11HG
	***ucqEA-A8-3***	A08	17.61	17.4–18	15.81	7.50	27.08	11GS
	*ucqEA-A8-4*	A08	21.91	20.8–23.6	10.59	5.65	11.10	11GS
	***ucqEA-A8-5***	A08	23.88	23.36–24.4	29.03–46.18	7.75–8.25	29.68–35.44	09DL/10DL/11HG
	***ucqEA-A8–6***	A08	25.91	25.76–26.05	38.61–57.54	7.32–9.11	28.88–45.05	11DL/12DL/13DL/11WH/12WH/13WH
	***ucqEA-A8-7***	A08	27.61	26.84–28.37	10.01–14.38	6.78–7.93	14.03–26.74	**10GS/11GS**
	***ucqEA-A8-8***	A08	28.80	28.57–29.04	39.60–50.33	7.99–8.59	31.50–40.00	**10DL/11DL/12DL**
	***ucqEA-A8-9***	A08	32.35	32.14–32.57	10.18–35.49	5.93–7.45	10.85–26.10	10DL/11DL/13DL/11WH/12WH/13WH/11GS
	***ucqEA-C3-1***	C03	175.74	174.98–176.5	5.17–32.15	3.09–8.18	2.70–29.11	10DL/13DL/11WH
	***ucqEA-C3-2***	C03	182.80	182.68–182.93	19.08–42.92	7.53–8.89	25.86–35.52	09DL/10DL/11DL/11GS
	***ucqEA-C3-3***	C03	183.48	183.15–183.81	6.80–26.83	6.83–7.77	15.82–28.59	11HG/10GS
	***ucqEA-C3-4***	C03	184.41	184.36–184.45	42.22–60.55	8.08–10.00	36.76–41.95	13DL/11WH/12WH/13WH
	***ucqEA-C3-5***	C03	185.23	185.04–185.42	16.96–50.81	7.24–9.10	23.78–37.87	09DL/10DL/11DL/12DL/11HG/11GS
	***ucqEA-C3-6***	C03	192.71	191.71–193.7	25.10–41.38	7.54–8.39	27.66–36.00	09DL/10DL/12DL/11WH/11HG/11GS
	*ucqEA-C3-7*	C03	194.71	190.8–202.3	6.65	6.90	17.57	10GS
	***ucqEA-C3-8***	C03	203.21	201.2–205.3	11.82	6.87	22.10	11GS
	*ucqEA-C9*	C09	74.81	72.8–76.6	3.51	−1.69	1.49	13WH
FAS	*ucqFAS-A8-1*	A08	14.11	14–16.1	10.81	−0.21	18.41	11GS
	***ucqFAS-A8-2***	A08	16.11	15.06–17.15	20.20–21.40	−0.18–0.17	19.31–21.36	**09DL/12DL**
	***ucqFAS-A8-3***	A08	21.91	21.44–22.37	27.00–31.71	−0.22–0.21	23.93–31.33	**12DL/13DL**
	***ucqFAS-A8-4***	A08	23.80	23.29–24.3	18.54–31.79	−0.26–0.19	21.23–28.64	09DL/10DL/11DL/11WH/11HG/11GS
	***ucqFAS-A8-5***	A08	28.35	28.08–28.63	10.11–35.61	−0.26–0.19	22.47–32.93	10DL/12DL/13DL/11WH/10GS
	***ucqFAS-A8-6***	A08	31.21	30.9–31.7	17.46	−0.25	26.43	11GS
	***ucqFAS-A8-7***	A08	32.31	31.87–32.74	22.99–28.94	−0.21–0.19	21.26–28.59	**10DL/13DL**
	***ucqFAS-A8-8***	A08	35.61	35.1–36	8.29	−0.19	20.06	10GS
	*ucqFAS-A9-1*	A09	21.91	19.3–23.9	6.17	0.09	4.37	12DL
	*ucqFAS-A9-2*	A09	25.41	23.6–25.8	4.59	0.08	3.39	11DL
	*ucqFAS-A9-3*	A09	28.91	27.99–29.82	4.70–5.61	0.08	3.44–3.61	**10DL/11DL**
	*ucqFAS-A9-4*	A09	34.51	33.54–35.47	4.46–5.89	0.08–0.10	3.78–4.61	10DL/11HG
	*ucqFAS-A9-5*	A09	41.11	37.6–41.3	3.97	0.09	4.13	11HG
	*ucqFAS-C2-1*	C02	44.51	42.8–45.8	4.05	−0.08	2.98	11DL
	*ucqFAS-C2-2*	C02	48.71	47.6–50	3.78	−0.07	2.42	10DL
	*ucqFAS-C2-3*	C02	51.01	49–51.5	3.78	−0.08	2.77	11DL
	*ucqFAS-C2-4*	C02	54.01	53.47–54.54	3.74–3.89	−0.07	2.48–2.74	**10DL/11DL**
	*ucqFAS-C2-5*	C02	56.51	56–56.8	6.77	−0.18	14.55	10GS
	*ucqFAS-C2-6*	C02	58.41	57.7–59	4.06	−0.07	2.59	10DL
	*ucqFAS-C2-7*	C02	60.01	59–60.1	5.36	−0.16	11.43	10GS
	*ucqFAS-C2-8*	C02	64.01	62.9–65.1	5.74	−0.08	3.93	13DL
	*ucqFAS-C3-1*	C03	110.51	108.2–112.5	4.20	−0.07	2.87	12DL
	***ucqFAS-C3-2***	C03	176.40	176.1–176.6	3.95–31.38	−0.28–0.10	2.65–30.92	09DL/10DL/11HG/11GS
	***ucqFAS-C3-3***	C03	182.80	182.76–182.85	5.63–31.15	−0.26–0.15	12.74–29.90	09DL/10DL/11DL/11WH/10GS
	***ucqFAS-C3-4***	C03	183.51	183.3–183.71	29.85–32.47	−0.22	26.88–29.00	**12DL/13DL**
	***ucqFAS-C3-5***	C03	185.51	185.36–185.66	13.83–32.11	−0.26–0.19	23.20–30.15	09DL/10DL/11DL/12DL/13DL/11WH/11GS
	***ucqFAS-C3-6***	C03	192.74	191.67–193.81	6.10–27.72	−0.26–0.13	4.09–26.43	10DL/11DL/12DL/13DL/11WH/10GS
	*ucqFAS-C5-1*	C05	108.67	107.46–109.88	3.74–4.37	−0.10–0.06	2.55–4.58	09DL/11HG
	*ucqFAS-C5-2*	C05	112.71	112.10–114.4	4.05	−0.09	4.22	11HG
	*ucqFAS-C5-3*	C05	116.71	116.32–117.09	4.10–4.50	−0.06	2.41–3.05	**09DL/10DL**
	*ucqFAS-C6*	C06	27.11	23.10–27.5	3.62	0.06	2.39	13DL
	*ucqFAS-C7*	C07	72.61	65.70–78.2	3.82	0.10	4.10	11GS
	*ucqFAS-C8*	C08	103.21	102.7–104.4	3.57	−0.08	2.47	11WH

a C-QTL, consensus QTL.

b Chromosome, the QTL located on.

c PV, Phenotypic variation the QTL explained (R2,%).

*The QTL bold represents the major QTL and the micrownvironments bold are corresponding to the environmental specific QTL.*.

In order to explore the pleiotropic QTL that simultaneously control multiple FA compositions, the 204 consensus QTL were then integrated into 127 unique QTL (Table 3). In the result, 2 unique QTL each simultaneously control 5, 6, and 7 different compositions, respectively. There were 20 QTL that simultaneously control two distinct compositions, 12 QTL simultaneously control three distinct compositions, and one QTL simultaneously control four different compositions, the rest of the unique QTL were composition-special and just control one composition for each.

**Table 3:** The revealed unique QTL in KN DH population for fatty aicd compositions.

**unique-QTL**	**Chromosome**	**Peak position**	**Confedence interval**	**LOD value**	**Additive effect**	**PV(R^2^;%)**	**Consensus-QTL**
uuqA1-1	A01	24.59	22.76–26.41	5.33–6.19	−1.94–0.31	2.56–11.02	ucqALA-A1
							ucqOA-A1-1
uuqA1-2	A01	34.11	30–34.5	6.25	−2.04	2.87	ucqOA-A1-2
uuqA2-1	A02	59.11	58.9–59.5	4.73	1.03	7.07	ucqLA-A2-1
uuqA2-2	A02	63.91	62.92–64.89	5.22–6.38	0.93–1.14	5.42–7.69	ucqLA-A2-2
uuqA2-3	A02	69.11	66.5–73.5	4.87	0.78	4.26	ucqLA-A2-3
uuqA3-1	A03	18.91	17.4–22.8	3.59	0.21	5.33	ucqALA-A3-1
uuqA3-2	A03	104.11	101–104.8	7.04	−0.33	8.60	ucqALA-A3-2
uuqA3-3	A03	109.3	108.19–110.42	3.44–6.89	−0.62–0.32	2.15–8.42	ucqLA-A3-1
							ucqALA-A3-3
uuqA3-4	A03	112.41	111.59–113.22	4.18–6.26	−0.22–0.16	4.39–6.94	ucqALA-A3-4
uuqA3-5	A03	115.81	115.3–116.5	5.58	−0.53	3.31	ucqLA-A3-2
uuqA3-6	A03	120.11	119–121.4	4.56	−0.19	5.13	ucqALA-A3-5
uuqA3-7	A03	127.71	126.1–129.2	4.40	−0.24	7.74	ucqALA-A3-6
uuqA4-1	A04	5.51	1.9–6.3	4.78	−1.77	2.15	ucqOA-A4
uuqA4-2	A04	69.61	68.6–71.4	3.56	0.08	1.75	ucqPA-A4
uuqA5-1	A05	12.91	12.5–14.5	3.76	−0.23	4.50	ucqALA-A5-1
uuqA5-2	A05	18.61	15.2–19.4	4.30	−0.17	5.14	ucqALA-A5-2
uuqA5-3	A05	23.91	22.7–26.8	7.29	−0.32	8.42	ucqALA-A5-3
uuqA5-4	A05	33.81	33.65–33.98	4.12–5.74	−0.29–0.24	4.55–7.31	ucqALA-A5-4
uuqA5-5	A05	40.84	40.3–46.2	4.34–5.70	−0.11	4.74–8.00	ucqALA-A5-5
uuqA5-6	A05	45.91	44–46.2	5.15	−0.21	6.74	ucqALA-A5-6
uuqA5-7	A05	49.31	48.6–50.9	5.75	−0.22	7.42	ucqALA-A5-7
uuqA5-8	A05	50.5	49.94–51.07	4.09–5.58	−0.05	4.43–6.56	ucqALA-A5-8
uuqA5-9	A05	57.81	57.4–59	3.88	−0.23	4.21	ucqALA-A5-9
uuqA6-1	A06	4.31	2–10.8	3.98	0.17	4.09	ucqALA-A6-1
uuqA6-2	A06	50.62	50.37–50.86	4.94–4.52	0.18–0.29	5.73–10.37	ucqALA-A6-2
uuqA6-3	A06	53.91	52.3–54.7	4.26	0.48	2.26	ucqLA-A6-1
uuqA6-4	A06	59.81	58.3–62.1	3.48	0.42	1.65	ucqLA-A6-2
uuqA6-5	A06	60.01	59.8–61.9	5.20	0.32	11.82	ucqALA-A6-3
uuqA6-6	A06	62.15	61.45–62.84	4.58–5.54	0.09–0.55	2.55–5.42	ucqALA-A6-4
							ucqPA-A6
							ucqLA-A6-3
uuqA6-7	A06	65.21	64.6–65.6	4.45	0.25	7.69	ucqALA-A6-5
uuqA6-8	A06	70.21	68–71.8	3.99	0.46	2.12	ucqLA-A6-4
uuqA6-9	A06	117.81	113.5–120.1	3.55	0.20	4.98	ucqALA-A6-6
uuqA7-1	A07	6.91	3.6–8.9	3.90	3.67	7.05	ucqEA-A7
uuqA7-2	A07	97.51	94.4–97.8	8.99	−0.21	5.69	ucqPA-A7-1
uuqA7-3	A07	106.91	106.6–107.6	5.73	−0.10	2.87	ucqPA-A7-2
uuqA7-4	A07	114.51	114.2–119.5	5.33	−0.09	2.73	ucqPA-A7-3
uuqA7-5	A07	118.11	114.5–124.2	3.93	0.19	4.92	ucqALA-A7
uuqA8-1	A08	6.01	2.6–11.2	5.83	4.57	6.23	ucqEA-A8-1
uuqA8-2	A08	11.61	10.08–13.13	10.29-19.45	−6.01–6.97	14.43–23.78	ucqOA-A8-1
							ucqALA-A8-1
							ucqEA-A8-2
uuqA8-3	A08	13.99	13.35–14.63	10.81–26.40	−1.61–1.34	18.41–25.97	ucqLA-A8-1
							ucqFAS-A8-1
uuqA8-4	A08	16.11	15.06–17.14	20.20–21.40	−0.18–0.17	19.31–21.36	ucqFAS-A8-2
uuqA8-5	A08	17.61	17.41–17.8	13.69–15.81	−1.75–7.5	23.78–27.08	ucqLA-A8-2
							ucqEA-A8-3
uuqA8-6	A08	19.78	19.43–20.14	6.08–30.80	−6.67–6.26	11.35–34.10	ucqOA-A8-2
							ucqLA-A8-3
							ucqALA-A8-2
uuqA8-7	A08	21.88	21.48–22.28	4.35–31.71	−3.34–5.65	7.34–31.33	ucqALA-A8-3
							ucqEA-A8-4
							ucqFAS-A8-3
uuqA8–8	A08	23.5	23.03–23.98	8.08–46.04	−1.84–1.56	10.00–35.36	ucqLA-A8-4
							ucqSA-A8-1
							ucqALA-A8-4
uuqA8-9	A08	24	23.78–24.23	18.54–47.97	−8.46–8.25	21.23–36.21	ucqFAS-A8-4
							ucqEA-A8-5
							ucqOA-A8-3
uuqA8-10	A08	25.91	25.81–26.01	17.10–57.54	−9.46–9.11	17.63–45.05	ucqPA-A8-1
							ucqOA-A8-4
							ucqEIA-A8-1
							ucqEA-A8-6
							ucqLA-A8-5
uuqA8-11	A08	27.69	27.35–28.02	10.01–25.82	−6.57–7.93	14.03–26.74	ucqEA-A8-7
							ucqSA-A8-2
							ucqOA-A8-5
uuqA8-12	A08	28.26	28.04–28.49	10.11–46.57	−0.3402–0.19	22.47–37.226	ucqPA-A8-2
							ucqFAS-A8-5
uuqA8-13	A08	28.8	28.66–28.94	6.91–50.33	−7.39–8.59	12.28–40.00	ucqEA-A8-8
							ucqLA-A8-6
							ucqALA-A8-5
							ucqOA-A8-6
uuqA8-14	A08	31.21	30.9–31.7	17.46	−0.25	26.43	ucqFAS-A8-6
uuqA8-15	A08	32.32	32.23–32.41	5.44–35.49	−1.89–7.45	9.09–32.05	ucqPA-A8-3
							ucqSA-A8-3
							ucqALA-A8-6
							ucqEIA-A8-2
							ucqFAS-A8-7
							ucqLA-A8-7
							ucqEA-A8-9
uuqA8-16	A08	32.9	32.67–33.14	22.43–29.84	−7.84–5.76	19.55–24.83	ucqOA-A8-7
uuqA8-17	A08	35.36	35.04–35.67	8.29–16.72	−7.65–0.19	20.06–26.54	ucqOA-A8-8
							ucqFAS-A8-8
uuqA8-18	A08	38.28	37.85–38.72	5.48–9.47	−0.36–0.27	8.52–18.47	ucqALA-A8-7
uuqA8-19	A08	42.01	41.74–42.21	3.96–7.65	−0.26–0.22	6.71–10.04	ucqALA-A8-8
uuqA8-20	A08	74.81	70.7–76.6	3.60	−0.52	1.72	ucqEIA-A8-3
uuqA9-1	A09	10.6	10.1–11.6	4.50	0.49	2.75	ucqLA-A9-1
uuqA9-2	A09	21.91	20.88–22.93	4.47–6.17	0.09–0.52	2.36–4.37	ucqLA-A9-2
							ucqFAS-A9-1
uuqA9-3	A09	24.31	23.9–24.8	4.02	−0.17	4.84	ucqALA-A9-1
uuqA9-4	A09	25.41	24.71–26.1	4.59–5.87	0.08–0.57	3.39–3.53	ucqLA-A9-3
							ucqFAS-A9-2
uuqA9-5	A09	28.91	27.99–29.82	4.70–5.61	0.07–0.08	3.44–3.61	ucqFAS-A9-3
uuqA9-6	A09	31.81	31.3–32.5	5.17	−0.19	6.14	ucqALA-A9-2
uuqA9-7	A09	34.62	33.71–35.53	4.46.79	−0.23–0.10	3.78–7.84	ucqFAS-A9-4
							ucqALA-A9-3
uuqA9-8	A09	41.11	37.6–41.3	3.97	0.09	4.13	ucqFAS-A9-5
uuqA9-9	A09	51.91	51.3–54.4	3.57	−0.24	4.88	ucqALA-A9-4
uuqA9-10	A09	63.61	62.9–66.1	6.30	−0.32	8.37	ucqALA-A9-5
uuqA9-11	A09	69.61	69.2–72.1	3.63	−0.25	4.98	ucqALA-A9-6
uuqA9-12	A09	90.91	89.5–100.7	3.78	−0.46	1.93	ucqLA-A9-4
uuqA9-13	A09	159.01	158.2–160	5.12	−1.03	7.67	ucqLA-A9-5
uuqA10-1	A10	15.61	15.4–16.3	4.01	1.04	3.05	ucqLA-A10
uuqA10-2	A10	53.81	52.9–54.4	5.92	−0.23	6.53	ucqALA-A10-1
uuqA10-3	A10	59.01	57.9–60.4	4.87	−0.20	5.59	ucqALA-A10-2
uuqA10-4	A10	70.6	69.04–72.15	5.92–6.97	−0.229–0.225	6.93–9.2	ucqALA-A10-3
uuqC1-1	C01	44.31	43.3–44.8	3.84	0.17	4.07	ucqALA-C1-1
uuqC1-2	C01	54.71	54.2–55.1	4.32	−1.31	7.79	ucqLA-C1
uuqC1-3	C01	57.91	57.1–60.9	3.51	0.16	3.51	ucqALA-C1-2
uuqC1-4	C01	78.81	78.5–80.2	5.46	−0.08	10.21	ucqSA-C1
uuqC2-1	C02	24.11	23.6–24.7	4.80	0.19	4.90	ucqALA-C2-1
uuqC2-2	C02	45	43.83–46.18	3.67–4.05	−0.08–0.18	2.98–3.79	ucqFAS-C2-1
							ucqALA-C2-2
uuqC2-3	C02	48.71	47.6–50	3.78	−0.07	2.42	ucqFAS-C2-2
uuqC2-4	C02	50.87	50.32–51.42	3.58–4.83	−0.08–0.21	2.77–5.57	ucqALA-C2-3
							ucqFAS-C2-3
uuqC2-5	C02	54.01	53.47–54.54	3.74–3.89	−0.074–0.072	2.48–2.74	ucqFAS-C2-4
uuqC2-6	C02	56.51	56.-56.8	6.77	−0.18	14.55	ucqFAS-C2-5
uuqC2-7	C02	58.41	57.7–59	4.06	−0.07	2.59	ucqFAS-C2-6
uuqC2-8	C02	59.89	59.43–60.35	4.05–5.36	−0.16–0.20	4.74–11.43	ucqALA-C2-4
							ucqFAS-C2-7
uuqC2-9	C02	64.01	62.9–65.09	5.74	−0.08	3.93	ucqFAS-C2-8
uuqC3-1	C03	25.91	25.6–26.3	51.10	2.41	35.53	ucqEIA-C3-1
uuqC3-2	C03	110.51	108.2–112.5	4.20	−0.07	2.87	ucqFAS-C3-1
uuqC3-3	C03	174	173.26–174.75	3.54–11.49	−3.22–0.18	2.75–12.95	ucqSA-C3-1
							ucqOA-C3-1
							ucqALA-C3-1
uuqC3-4	C03	175.8	175.13–176.46	5.17–32.15	−0.07–8.18	2.70–29.11	ucqEA-C3-1
							ucqSA-C3-2
uuqC3-5	C03	176.4	176.18–176.61	3.95–32.10	−2.69–0.10	1.88–30.92	ucqLA-C3-1
							ucqFAS-C3-2
							ucqOA-C3-2
uuqC3-6	C03	178.06	176.85–179.28	4.15–5.20	−0.29–0.19	5.13–9.68	ucqALA-C3-2
uuqC3-7	C03	182.8	182.75–182.84	5.63–42.92	−9.33–8.89	12.74–36.97	ucqOA-C3-3
							ucqEA-C3-2
							ucqFAS-C3-3
uuqC3-8	C03	182.84	182.66–183.02	8.67–34.15	−1.77–1.44	16.23–30.74	ucqLA-C3-2
uuqC3-9	C03	183.4	183.3–183.51	6.80–40.15	−8.02–7.77	9.08–35.62	ucqSA-C3-3
							ucqALA-C3-3
							ucqEIA-C3-2
							ucqOA-C3-4
							ucqEA-C3-3
							ucqFAS-C3-4
uuqC3-10	C03	184.41	184.38–184.43	9.44–60.05	−10.02–10.00	20.11–41.95	ucqPA-C3-1
							ucqSA-C3-4
							ucqLA-C3-3
							ucqEIA-C3-3
							ucqEA-C3-4
							ucqOA-C3-5
uuqC3-11	C03	185.35	185.26–185.44	4.31–50.81	−9.05–9.10	4.96–37.87	ucqLA-C3-4
							ucqEA-C3-5
							ucqOA-C3-6
							ucqALA-C3-4
							ucqFAS-C3-5
uuqC3-12	C03	190.91	190.3–191.51	23.24–33.73	−1.90–1.47	23.57–30.74	ucqLA-C3-5
uuqC3-13	C03	192.58	192.11–193.04	3.55–41.38	−9.06–8.39	3.50–36	ucqOA-C3-7
							ucqSA-C3-5
							ucqEIA-C3-4
							ucqEA-C3-6
							ucqFAS-C3-6
							ucqLA-C3-6
							ucqALA-C3-5
uuqC3-14	C03	194.71	192.81–196.6	6.65–21.77	−6.96–6.90	17.57–22.39	ucqPA-C3-2
							ucqOA-C3-8
							ucqEA-C3-7
uuqC3-15	C03	203.21	201.77–204.64	11.00–11.82	−1.56–6.87	19.03–22.10	ucqLA-C3-7
							ucqEA-C3-8
uuqC4-1	C04	120.51	116.3–123.5	3.41	0.23	6.11	ucqALA-C4
uuqC4-2	C04	124.31	123.9–129	3.78	0.14	6.67	ucqPA-C4
uuqC5-1	C05	24.01	21.4–37.6	3.63	0.87	8.94	ucqEIA-C5
uuqC5-2	C05	47.01	40.4–53.9	5.62	0.32	9.95	ucqALA-C5-1
uuqC5-3	C05	55.18	51.88–58.48	3.69–8.02	0.25–0.56	3.07–14.41	ucqLA-C5-1
							ucqALA-C5-2
uuqC5-4	C05	63.85	62.6–65.11	5.49–9.84	0.26–0.32	12.00–17.24	ucqALA-C5-3
							ucqALA-C5-4
uuqC5-5	C05	71.48	70.61–72.34	5.85–7.75	0.23–0.33	9.64–18.20	ucqALA-C5-5
uuqC5-6	C05	104.01	101.5–105	3.44	−0.51	2.61	ucqLA-C5-2
uuqC5-7	C05	108.67	107.46–109.88	3.74–4.37	−0.10–0.06	2.55–4.58	ucqFAS-C5-1
uuqC5-8	C05	110.74	110–111.49	4.04–4.88	−0.48–0.44	1.99–2.20	ucqLA-C5-3
							ucqLA-C5-4
uuqC5-9	C05	112.71	112.1–114.4	4.05	−0.09	4.22	ucqFAS-C5-2
uuqC5-10	C05	115.21	114.4–116.9	4.03	−0.44	1.78	ucqLA-C5-5
uuqC5-11	C05	116.79	116.44–117.14	4.10–5.08	−0.47–0.07	2.29–3.05	ucqFAS-C5-3
							ucqLA-C5-6
uuqC5-12	C05	129.41	125.8–135.1	4.84	−0.09	11.95	ucqPA-C5
uuqC6-1	C06	27.11	23.1–27.5	3.62	0.06	2.39	ucqFAS-C6
uuqC7-1	C07	64.11	61.9–65.3	3.41	0.22	3.99	ucqALA-C7-1
uuqC7-2	C07	72.61	69.36–75.85	3.82–5.77	0.10–0.28	4.10–6.57	ucqALA-C7-2
							ucqFAS-C7
uuqC8-1	C08	95.61	91.3–97.3	3.52	−0.23	3.94	ucqALA-C8-1
uuqC8-2	C08	103.21	102.7–104.4	3.57	−0.08	2.47	ucqFAS-C8
uuqC8-3	C08	105.91	104.8–111	3.68	−0.24	4.12	ucqALA-C8-2
uuqC9-1	C09	19.01	17.8–19.2	5.88	−0.23	6.84	ucqALA-C9-1
uuqC9-2	C09	21.91	21–23.2	5.42	−0.22	6.35	ucqALA-C9-2
uuqC9-3	C09	64.07	62.87–65.28	3.53–6.95	0.04–1.92	1.52–6.34	ucqSA-C9-1
							ucqOA-C9-1
uuqC9-4	C09	74.81	72.83–76.78	3.51–5.65	−1.69–2.29	1.49–5.74	ucqSA-C9-2
							ucqOA-C9-2
							ucqEA-C9
uuqC9-5	C09	184.41	184.3–184.5	54.99	2.61	40.53	ucqEIA-C9

*The “uuq” initiated QTL represent the unique QTL and “cuq” initiated QTL represent the corresponding consensus QTL of these unique QTL.*.

### Conditional QTL analysis for FA composition

To further explore the genetic relationship of closely correlated compositions at individual QTL level, the conditional QTL analysis was also carried out. When the phenotypic data of the eight compositions were conditioned with each other, a total of 3037 conditional identified QTL were found, and the PV value ranged from 1.32 to 39.75%, the average value were significantly reduced to 7.40% compared to 17.99% of the unconditional analysis (Table S7). Unlike the unconditional result, these conditional identified QTL were more evenly distributed on the 19 linkage groups of rapeseed and the total occupied a proportion on A8 and C3 linkage groups that reduced to about 22.13% [(266 + 406)/3,037] compared to 67.73% of proportion under unconditional analysis. For the single composition, there were 152, 147, 507, 522, 528, 132, 546, and 503 QTL, respectively for C16:0, C18:0, C18:1, C18:2, C18:3, C20:1, C22:1, and FAS (Table S7).

These conditional identified QTL were subsequently integrated into 2241 conditional consensus QTL by meta-analysis (Table S8). Of the 2241 conditional consensus QTL, 288 were detected to be expressed in two microenvironments, 105 were simultaneously expressed in three microenvironments, 40 could be detected to be expressed in four microenvironments. Additionally, 14, 11, 13, 1, 1, and 2 QTL were respectively expressed in 5, 6, 7, 8, 9, and 10 microenvironments, and the remaining were specially expressed in only one microenvironment. For each composition, there were 145, 136, and 124 conditional consensus QTL revealed for the three compositions of C16:0, C18:0, and C20:1, respectively. Furthermore, there were more than 300 consensus QTL each for five compositions (C18:1, C18:2, C18:3, C22:1, and FAS), the most was 395 for C22:1 (Table S8). In addition, remarkable different detection of the major QTL for each composition was discovered after the conditional analyses. There were 4, 18, 42, and 27 novel major QTL revealed respectively for C18:0, C18:1, C18:3, and FAS, and the others contained the overlapped confidence with the QTL of unconditional results. Surprisingly, although there were 3, 30, 5, and 35 novel discovered major QTL respectively for C16:0, C18:2, C20:1, and C22:1, the major QTL that was detected in the unconditional analyses for the four compositions was absent within the conditional analyses. These results suggested that many unconditional QTL for some traits were contributed by other traits rather than attributed directly to themselves.

### Epistatic interaction analysis for the metabolic processes of FA composition

In addition to additive QTL, epistatic interaction was also considered as an important contributor for the genetic basis of PV in crops (Jourdren et al., 1996). FA components of oil seed crops typically showed the quantitative composition feature and the complex epistatic interaction usually played a vital role in the metabolic processes and finally determined the quality of the edible oil (Wang et al., 2015). A total of 69 pairs of additive × additive (AA) type epistatic QTL interaction were observed, the additive effect value ranged from −4.56 to 4.36 and the number of the epistatic QTL pairs for single composition varied from 2 of C16:0 to 17 of FAS (Table S3). Notably, the distribution of these epistatic QTL displayed a large uneven distribution pattern among the 19 linkage groups. In general, for the 69 interaction pairs, 49 pairs of QTL interaction (accounting for 71% of the proportion) were obtained from the interaction between A8 and C3, and for each linkage group contained 56 and 54 involved QTL, respectively. There were 8, 5, 3, and 2 QTL interaction loci detected for the linkage group of C5, A9, C2, and A6, respectively. In addition, only one QTL interaction loci was observed on A3, A5, A10, C1, C7, and C9. As for the environmental distribution of these epistatic interaction QTL, 31 pairs of epistatic QTL were detected in the winter type, 30 pairs were detected in the semi-winter type and the remaining 8 epistatic pairs belong to the spring type (Table S3).

### Detection of lipid metabolism related genes in CI of QTL

**Figure 3:**
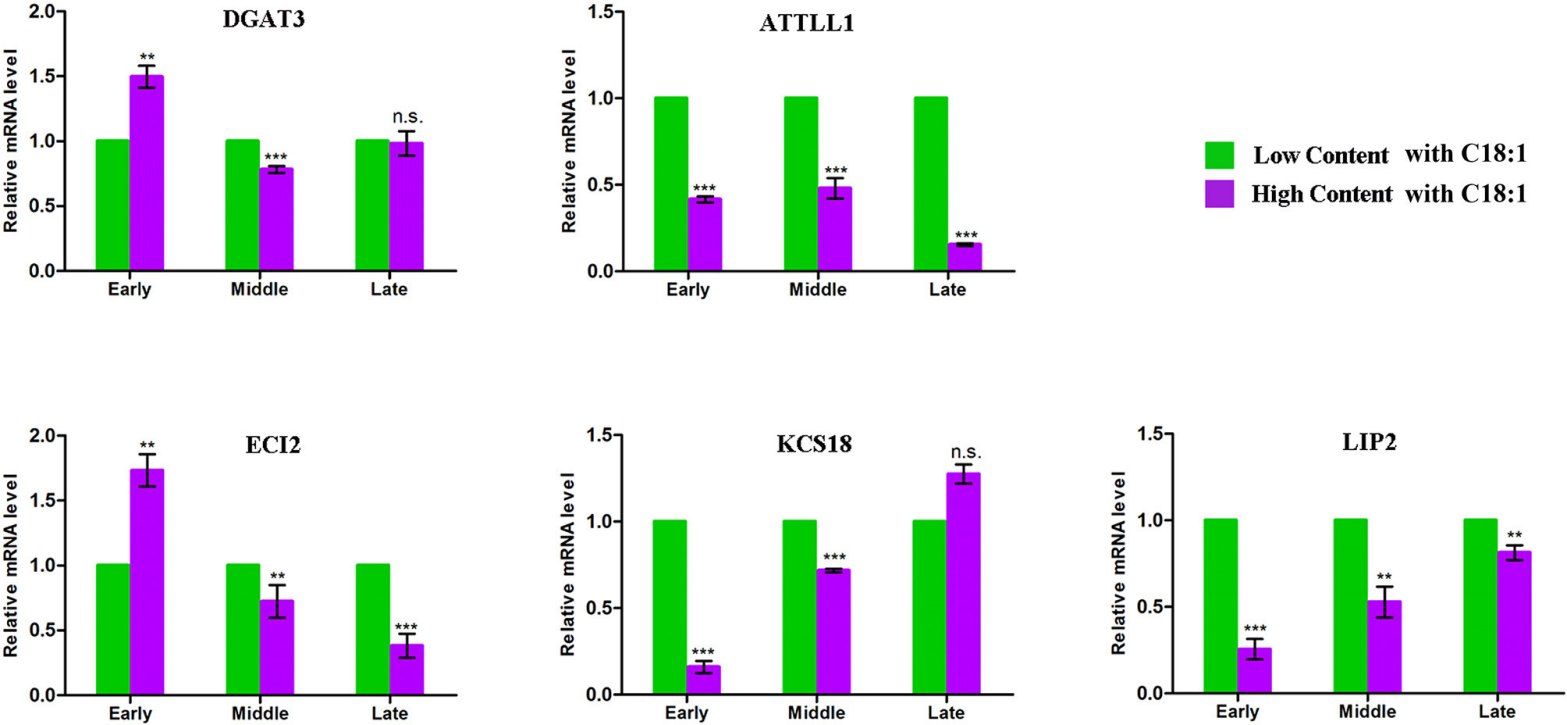
qPCR analysis for the five acyl lipid metabolism related candidates. The x-axis indicates the three different developmental stages of seed and y-axis indicates the mRNA expression level of each candidate gene. The green represents the material contains low C18:1 concentration and the purple represents the material contains high concentration of C18:1. Early indicates that the materials were collected at the stage of 15 days after flowering, middle indicates the stage of 30 days after flowering and late indicates the stage of 45 days after flowering. The expression of each gene were calculated based on three biological replicates. Bar represent the standard deviation calculated by ΔΔ Ct method. **Significant at *p* < 0.01, *** Significant at *p* < 0.001, n.s. No significance *p* > 0.05.

Using the comparative genomics approach together with the released genome sequence of *B. napus* (Chalhoub et al., 2014), more than thirteen thousand homologous genes of *Arabdopsis* underlying the CI of the 91 unique QTL were observed (Table S4). 68 genes were further filtered out relate to the acyl-lipid metabolism. Exclusion of seven genes that had no sequence difference in the parents, 61 orthologous genes were left and these potential candidates were involved in several main processes of FA metabolism, including Plastidial FA Synthesis, FA Elongation, Lipid Trafficking, TAG Synthesis/Degradation, β-Oxidation and oilbody formation (Table S5). Noteworthy, seven genes (including *DGAT3, ATTLL1, ECI2, LIP2, DGT2*, and *KCS17/18*) that located in the major QTL region with large PV were also identified. For example, the FA elongation related gene *KCS18* were simultaneously involved in seven major QTL of ucqEA-A8-6, ucqEA-C3-3, ucqOA-A8-4, ucqPA-A8-1, ucqEIA-A8-1, ucq-EIA-C3-2, and ucqFAS-C3-4, with the largest explained phenotypic variation value of 45.05%. In order to validate the potential essential role of these seven major QTL located genes, five of them which respectively belonged to the five different processes of acyl lipid metabolism, were selected for the qPCR analysis (Figure 3). The embryo of three developmental stages after flowering time, with low and high content of C18:1, were used for qPCR assay. It was revealed that with the development of the seed, the expression level of two genes *LIP2* and *KCS18*, which respectively belonged to the processes of plasitdial FA synthesis and FA elongation, became more and more stronger in the material with high content of C18:1 compared with the material with low C18:1 content. The expression level of *DGAT3* (the TAG synthesis related gene) in the material with high C18:1 content was higher than that of the material with low C18:1 content, while with the development of the seeds went on, its expression level tended to be identical in the two different types of materials. In addition, the expression levels of two TAG degradation and β-Oxidation related genes, *ATTLL1* and *ECI2*, became gradually weaker in the material with higher C18:1 content than that of the material with lower C18:1 content. Together, these results suggested that these candidates closely related to FA metabolism during the seed development of rapeseed.

### Implementation of network interaction analysis among candidates and construction of potential regulatory network involving metabolic processes of FA in *B. napus*

**Figure 4:**
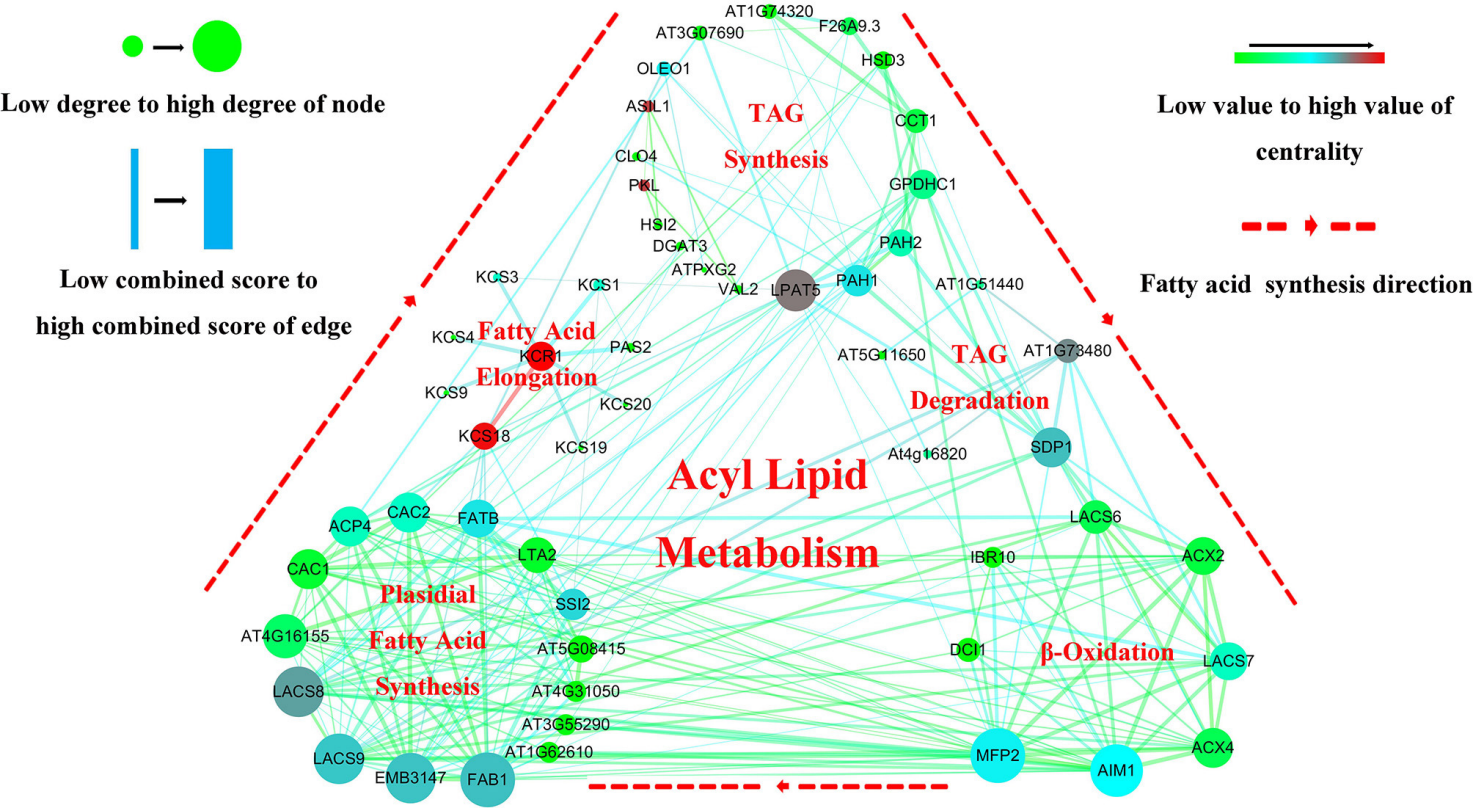
Gene interaction network analysis associated with the acyl lipid metabolism related candidates underlying the CI of unique QTL. The visualized interaction network of 54 candidates was constructed by String software and exhibited by Cytoscape V-3.5.0 software. The five different regions represent different metabolic pathway of acyl lipid metabolism. Nodes represent the potential candidates and edges represent the interaction of them. Node size represents “Degree” and edge size represents “Combined-score,” and the color for nodes and edges represents the “Betweenness certrality” and “Edge Betweenness”, respectively. All the value for these four parameters was calculated by Network Analyzer that included in Cytoscape V-3.5.0 software.

**Figure 5:**
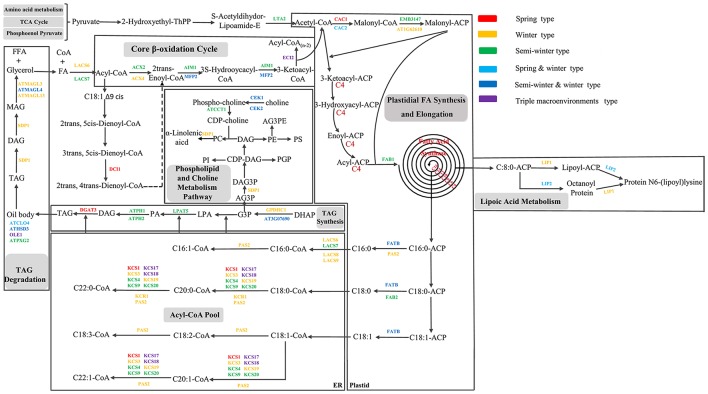
Potential regulatory network and some candidate genes associated with acyl lipid metabolism in *B. napus*. All the candidate genes used in the network originated from the CI of unique QTL of the seven FA compositions. Different colors with the genes indicated the candidates located in different consensus QTL associated with different breeding environment. The dashed line indicates that multiple reactions in this step might exist.

To further dissect the genetic mechanism of acyl lipid metabolism, 54 of the 61 candidates were linked and an interaction network based on the ortholog annotation in *A. thaliana* was constructed. The network contained 54 nodes and 221 edges and involved five lipid metabolic processes of TAG synthesis/degradation, FA elongation, Plastidial FA synthesis and β-Oxidation (Figure 4). The network result showed that despite these interacted candidates attributed to different metabolic processes, the complex interaction, direct or in direct, still closely existed among them. Some predominant candidates, such as *FAB1, LPAT5* and *MFP2* extensively interacted with other genes, this suggested the essential role for them in the process of acyl-lipid metabolism. Although the acyl-lipid metabolism process was characterized in *Arabidopsis* in detail (Li-Beisson et al., 2013), the similar work was seldom carried out in *B. napus* owing to its more complex genomic structure. To obtain a better understanding of the genetic basis of the FA formation and accumulation during seed development, the 61 specially selected orthologous candidates were used to construct an elaborated pathway related to the FA metabolism in *B. napus* (Figure 5). This main pathway contained several associated metabolism processes, including plastidial FA synthesis, FA elongation, TAG synthesis and degradation, phospholipid and sulfolipid synthesis, beta-oxidation, lipid trafficking and oil body formation.

A total of 15 orthologous genes were detected related to plastidial FA synthesis process. For example, short-chain dehydrogenase/reductase (SDRD) and *AT1G62610*, two orthologous candidates of *Arabidopsis* that were respectively involved in the peroxisomes metabolism and the Ketoacyl-ACP Reductase activity, jointly controlled the three FA compositions of C18:2, C18:3 and C16:0 (Quan et al., 2013), which were positioned in the same QTL of ucqLA-A6-2. Long-Chain acyl-CoA synthease, *LACS8* and *LACS9*, two stearyl coenzyme A synthease catalyzing the formation of stearyl Co-A from stearic acid, were detected in ucqALA-A10-2 and ucqALA-A10-1, respectively. As one of the most committed step in the FA biosynthesis process, the conversion of acetyl-CoA to malonyl-CoA is catalyzed by the acetyl carboxylase (*ACCase*) (Baud et al., 2003). Two *ACCase* subunits of *BCCP1* and *CAC2* were detected in the CI of ucqALA-A3-1, ucqLA-A9-5 and ucqEIA-C5 respectively, and probably control the biosynthesis of three FA compositions of C18:2, C18:3, and C20:1. The plastid E2 subunit of pyruvate dehydrogenase of *PLE2*, which played a great important role in the early embryonic development of *Arabidopsis* (Lin et al., 2003), was observed in the CI of ucqALA-A9-6 with a negative additive effect.

The *de-novo* biosynthesis process of FA in plastid is sequentially accompanied by the elongation of acyl-CoA in the endoplasmic reticulum (Li-Beisson et al., 2013). In this study, a total of 10 potential candidates involved in FA elongation were identified in the CI of some consensus QTL. Among these candidates, eight *KCR* family genes were identified containing *KCR*1/3/4/917/18/19/20. It is noteworthy that *KCR17* and *KCR18* were respectively located in ten and seven major QTL (Table S5). Additionally, *KCR1* and another potential candidate *PAS2* that were involved in the FA elongation process were also found in the QTL of ucq-ALA-A7 and ucqALA-A10-1, respectively.

The biosynthesis process of TAG typically contains four main sequential steps and was catalyzed by various functional genes (Ohlrogge and Browse, 1995; Beisson et al., 2003). Six genes participating in this process were revealed. For example, *GPDHC1*, a gene encodes a protein with NAD-dependent glycerol-3-phosphate (*G3P*) dehydrogenase activity was found in the conversion step of *DHAP* to *G3P* and underlying the CI of ucqALA-A5-2. Lysophosphatidyl acyltransferase (*LPAT*) family contains five members of *LPAT1* to *LPAT5*, all of which possesses the main function in the conversion of lysophosphatidic acid to phosphatidic acid (Beisson et al., 2003). In this reaction step, *LPAT5* was revealed in the CI of ucqSA-C1 with a negative additive effect. The following reaction of the process mentioned above was the dephosphorylation of PA that was catalyzed by phospatidate phosphatase (*PP*) to form DAG. PP played a pivotal role in this step and its activity was also closely related to two *Arabidopsis* homologs phosphatidic acid phosphohydrolase of *PAH1* and *PAH2*, both of which possesses Mg^2+^-dependent PP activity when expressed in yeast and were strongly expressed in the developing seeds (Eastmond et al., 2010) and were detected in the CI of ucqLA-C5-2 and ucqALA-A6-6, respectively. The final step of triacylglycerols biosynthesis was the acylation reaction that utilizing DAG to form TAG. Several important genes involving this step were found in previous studies, such as diacylglycerol acyltransferase *(DGAT)* family genes of *PDAT* and *PDCT* (Lardizabal et al., 2001). Besides the role of forming of TAG, DAG also was used to generate phosphatidylcholine together with CDP-choline, which was generated through a two-step sequential reaction conversion of choline to phospho-choline and phospho-choline to CDP-choline. Two potential candidates of choline kinas, *CK1* and *CK2*, underlying the first conversion process and one candidate of phosphorylcholine cytidylyltransferase 1 (*CCT1*) underlying the second were revealed and located in the ucq-ALA-A7, ucqPA-A7-3, and ucqALA-C8-1, respectively.

TAG biosynthesis occurs at the ER and once synthesized TAG molecules will coalesce to form the specific structure of oil bodies or lipid droplets. These organelles consist of a TAG core surrounded by a number of different proteins and the most abundant of these proteins are the oleosins (Jolivet et al., 2004). Here, *OLE1* and *CLO4* were detected in five QTL of ucqALA-A3-5, ucqALA-A7, ucqALA-C7-2, ucqLA-A2-2 and ucqFAS-C7. Besides the ER, the biosynthesis of TGA probably also involves some reactions at the oil body (Huang, 1992). *A. thaliana* homologous genes of peroxygenase 2 (*ATPXG2*) and hydroxysteroid dehydrogenadse 3 (*HSD3*), two genes related to the formation of oil body were found and were associated with three QTL of ucqLA-A10, ucqALA-A3-4 and ucqALA-C7-1.

Seed stored oil mobilization via TAG degradation and β-oxidation processes contain series of sequential reactions and involves various catalyzing genes (Graham, 2008). Triacylglycerol undergoes three successive deacylation reactions to release free FA and glycerol in cells. Several candidates were revealed in these processes, for example, three potential Monoacylglycerol lipase (*MAGL*) family genes *MAGL3, MAGL4*, and *MAGL13*, both of which function in catalyzing the MAG to form free FA and glycerol, were detected in the CI of our QTL analysis.

Free FA and glycerol, both of which derived from the terminal degradation of TAG, were transported by an ABC related transporter of CTS into peroxisome and underlying a series of reactions to form CoA and FAs at first (Footitt et al., 2002). Before the core β-oxidation pathway occurs, CoA and FAs were converted into acyl-CoA by long chain acyl-CoA synthetase (*LACS*). In this step, two potential candidates *LACS6* and *LACS7* were found and associated with three QTL of ucqLA-C5-5, ucqFAS-C3-1, and ucqLA-A6-4. FA β-oxidation of high plants is a ubiquitous process that occurs in peroxisome and the core pathway contains four sequential reaction steps. First step is the conversion of Acyl-CoA to 2trans-Enoyl-CoA and catalyzed by acyl-CoA oxidase (*ACX*). In this step *ACX2* and *ACX4* were detected and located into ucqALA-C9-2 and ucqLA-A9-4, respectively. The second and third steps involve the conversion of 2trans-Enoyl-CoA to 3S-Hydrooycacyl-CoA next to 3-Ketoacyl-CoA, and these two steps are catalyzed by the same enzyme of multifunctional protein (*MPF*). The orthologous *MPF2* was revealed in this two steps and located in the two QTL of ucqFAS-C3-1 and ucqFAS-C5-2. Additionally, gene abnormal inflorescence meristem 1 (*AIM1*), which played an essential role in wound-induced jasmonic acid formation in peroxisome via FA β-oxidation pathway (Delker et al., 2007), was also detected in the QTL analysis and mapped into the ucqLA-A6-1.

Besides the potential FA metabolism related genes discussed above, two lipid trafficking genes *TGD2* and *PTAC4* were also revealed and associated with five QTL of ucqALA-C5-5, ucqFAS-C2-3, ucqFAS-C2-7, ucqALA-C2-3 and ucqALA-C2-4. *TGD2*, the phosphatidic acid-binding protein, played an important role in the process of polar lipid flipping across the ER and outer and inner chloroplast envelope membranes.

## Discussion

### Phenotypic variation and correlation analysis among different FA compositions

Quantitative compositions of various crops are genetically controlled by abundance of reciprocal genes and are generally affected by the surrounding environment (Si et al., 2003). The distribution patterns of C16:0, C18:0, C18:2, C18:3, and FAS displayed the typical normal or near-normal distribution, while for the three MUFAs, C18:1, C20:1, and C22:1, they totally showed double main peaks performance. This result was similar to the previous reports (Wang et al., 2015) despite the different populations used and it indicated that a few genes with major effect controlling MUFAs existed. Besides the similar distribution pattern of the composition content among these compositions, they also displayed remarkable correlation with each other. For example, due to the coherent biosynthetic process and the similar required catalytic enzymes, such as the KCS gene family, the two very long-chain FAs of C20:1 and C22:1 showed close positive correlation. This notion also could be considered as the cause for the close positive correlation among the six remaining compositions. While, owing to the competitive relationship of the same substrate for C18:1-CoA or the potential deviational regulation, C20:1 and C22:1 were all negatively correlated with the other six compositions.

### Unconditional and conditional QTL mapping analysis of FA

Generally, achievement of detecting accurate and reliable QTL depends on the large mapping population that holds high density genetic linkage map and multiyear and multisite field trials in crops (Asíns, 2002). In this study, a DH population that contained 348 lines and tested in 14 environments was utilized to perform the unconditional and conditional QTL mapping analysis. In total, 406 QTL were detected and unevenly distributed into the entire genome of *B. napus*, and 67.76% of them were found in two linkage groups of A8 and C3 (Figure S2). The positions for the majority of the detected QTL (99.15% for A8 and 98.55% for C3) of the two linkage groups were revealed into the coherent range of 2.6–42.3 and 171.9–206.9, with the mean CI of 1.5 and 3.0, respectively (Table S2). The feature of clustered distribution suggested abundant variations related to FA metabolism on A8 and C3.

For QTL mapping of FA, KN DH population is a new population that derived from the cross of two parents with a large difference in oil content and FA (Table 1; Wang et al., 2015), and have a big population number. Therefore, in present study, combining the high density linkage map, some novel QTL for some FA compositions were discovered by comparing to the previous studies (Burns et al., 2003; Hu et al., 2006; Zhao et al., 2008; Smooker et al., 2011; Yan et al., 2011; Wang et al., 2015). Three novel consensus QTL of ucqPA-C5, ucqSA-C1 and ucqEIA-C5 were revealed controlling three compositions, C16:0, C18:0 and C20:1, explaining PV value of 11.95, 10.21, and 8.94%, respectively. 12 novel consensus QTL of ucqALA-A3-1/2/3/4/5/6 and ucqALA-A9-1/2/3/4/5/6 were detected responsible for C18:3 with the largest PV value of 8.60%. Furthermore, 10 FAS related novel consensus QTL of ucqFAS-C2-1/2/3/4/5/6/7/8, ucqFAS-C7 and ucqFAS-C8 were also found and distributed in the three linkage groups of C2, C7 and C8 and with the largest PV value of 14.55% (Table S2).

In addition, some QTL reported in previous studies were finely divided into multiple QTL with smaller confidence interval in the present study. For example, Burns et al. (2003) detected some QTL that were distributed in the N8 linkage group respectively controlling six compositions of C16:0, C18:0, C18:1, C18:2, C20:1, and C22:1, both of which were divided into at least two consensus QTL and at most nine QTL for C22:1. In another study, Yan et al. (2011) applied a mapping population contains 451 markers to detect some FA compositions QTL located in the N8 linkage group and were involved in five FA compositions, C16:0, C18:1, C18:2, C20:1, and C22:1, the QTL for each composition were divided into more than one consensus QTL in this study; one N10 located QTL, responsible for the composition of C18:3 was also divided into two regions. Smooker et al. (2011) revealed two QTL that controlled the two compositions of C18:1 and C18:3 and were located in two linkage groups of A1 and C2, both of which were also detected in our result and divided into two consensus QTL. These results demonstrated that genetic linkage map with high marker density could facilitate accurately and meticulously the detection of QTL of interest and this is also prerequisite for the study of candidate detection.

Previous studies reported that the three very long chain unsaturated FA compositions, C22:1, C18:1, and C18:2, mainly composed seed oil in *B. napus* (Zhao et al., 2008; Smooker et al., 2011). In this study, the co-localization for some QTL which simultaneously controlled two distinct traits were also observed, and which were located on three sets of linkage groups of A8, A9, C3 and C5, A8 and C3, A8 and C3, respectively (Figure 2; Table S6). This indicated that the detection result of FA QTL in this study was reliable and was also consistent with the previous detection of which concentration of the three compositions was positively correlated with oil content in rapeseed (Malosetti et al., 2013).

The field trials that implemented here associated with successive seven years in three distinct breeding macroenvironments of winter type, semi-winter type and spring type. In general, the existence of quantitative trait is easily affected by the surrounding environment of breeding site (Malosetti et al., 2013), and multiple years and sites' field trial is of great significance for detecting the environmental stable and specific QTL. Some major QTL, which were successively detected in multiple macroenvironments, such as ucqEA-A8-9, ucqALA-C3-4, and ucqOA-C3-7, were considered as environment stable ones (Table 2). 22 macroienvironment specific QTL were revealed containing six semi-winter type QTL, two spring type QTL and 14 winter type QTL. The six semi-winter type QTL ucqPA-A8-3, ucqPA-C3-1, ucqSA-A8-3, ucqSA-C3-3/4, and ucqLA-C5-1 were detected in two years of 12WH and 13WH. ucqLA-A2-2 and ucqEA-A8-7 were the two spring type QTL and were detected in the two consecutive years' field trial of 10GS and 11GS. ucqOA-A8-6, ucqLA-C5-4, ucqALA-A3-4, ucqALA-A6-2, ucqALA-A10-3, ucqALA-C5-2, ucqEA-A8-8, ucqFAS-A8-2/3/7, ucqFAS-A9-3, ucqFAS-C2-4, ucqFAS-C3-4, and ucqFAS-C5-3 represented the winter type QTL and expressed in at least two successive years from 09DL to 13DL (Table 2). Majority of these environmental specific QTL were also major ones and will facilitate us to breed the cultivars that grow in specific environment.

In the light of the complicated interaction, using of unconditional QTL mapping method only could not completely dissect the genetic mechanism of quantitative traits in crops because of the direct correlation between two compositions on the individual level (Wen and Zhu, 2005). When the eight compositions were conditioned with each other, a mount of conditional QTL were revealed for each conditioned composition. These conditional QTL, identified or consensus, generally had similar additive effect value compared to the unconditional results, which suggested that the FA compositions were controlled by numerous of QTL with micro genetic effect that could not be detected under the unconditional condition. These conditional QTL were more evenly distributed in the 19 linkage groups of *B. napus* rather than the cluster distribution pattern on A8 and C3 linkage groups of the unconditional results. Through comparison of the detected QTL between the two different conditions, it was revealed that QTL could be divided into four types. The first type containing these QTL could be detected under the unconditional analysis but not in the conditional result. These types of QTL represented their actual attribution by other correlated composition and it might indicate some genes closely linked in the same loci and control the PV for distinct FA compositions. For example, two major unconditional consensus QTL of ucqPA-A8-3 and ucqALA-A6-2 were detected in two microenvironments respectively but couldn't be detected when conditioned on other seven compositions, which suggested that the two major QTL for the two compositions of C16:0 and C18:3 were in fact attributed by other compositions. The second type of QTL was those that could only be detected in the conditional result rather than the unconditional analysis, this suggested that the expression of some QTL with micro genetic effect were suppressed when simultaneously analyse two distinct traits but they appeared when the related compositions were conditionally eliminated. Taking the conditional consensus QTL of ccqEA/FAS-C2-2 for example, it could be detected in six microenvironments and with a small additive effect value of −0.08–0.05 while couldn't be detected under the unconditional condition. This result indicated that this QTL for C22:1 was covered by other compositions and couldn't be revealed when the unconditional analysis was performed. The third and fourth types of QTL were those that could simultaneously be detected under the two different conditions and the difference was that the former with slight change in the additive effect value and the latter showed that these conditional QTL possessed obvious change in additive effect but still had significant effect compared with the unconditional counterparts. These two types of conditional QTL commonly represent the QTL that with multiple function in controlling more than one compositions of their metabolic mechanism. The third type displayed some compositions controlled by some QTL which were independent of other compositions. For example, the major unconditional consensus QTL ucqSA-C3-3 was detected in two microenvironments of 12WH/13WH and with the additive effect value of−0.09–0.08. Two corresponding conditional QTL of ccqSA/PA-C3-3 and ccqSA/LA-C3-2 were detected when C18:0 was conditionally eliminated on the compositions of C16:0 and C18:2, and with the small additive effect value−0.11 and−1.01, respectively. This result demonstrated that the existence of QTL ucqSA-C3-3 was at least independent from the contribution of C16:0 and C18:2. For the fourth type of these QTL, a proper example was the major QTL ucqALA-C3-4 simultaneously detected in eight microenvironments and with a small additive effect value of−0.40–0.17, but when the composition of C18:3 was conditioned on composition of C22:1, the conditional consensus QTL ccqALA/EA-C3-9, the corresponding QTL of ucqALA-C3-4, was also detected in six microenvironments while the additive effect value increased to 4.13–7.89, this suggested that the composition of C18:3 were controlled not only by the QTL of the individual level but also partly attributed by the influence from C22:1. Another example is the consensus QTL ucqEA-A8-9, which represented a major QTL of C22:1 and was revealed in seven microenvironments with a large additive effect value of 5.93-7.45, while conditioned on the composition of C18:2, the additive effect value for the corresponding conditional QTL of ccqEA/LA-A8-1 greatly reduced to−0.48, this indicated that the genetic effect of ucqEA-A8-9 for C22:1 was largely contributed by C18:2. Together, conditional QTL analysis provides an excellent method to explore the genetic mechanisms for quantative traits, such as FA composition, that generally contain two effects of pleiotropism and close linkage, of crops in future.

### Epistasis analysis and underlying FA related candidate genes analysis

QTL mapping was an effective assay method for the functional study of the complex QTL in plants (Maughan et al., 1996). Besides the additive effect QTL, the epistasis interaction usually acted as an influential factor for the PV of some important agronomic traits in crops. Epistasis analysis in plants was reported decades ago and was considered as a genetic interaction at multiple loci (Richey, 1942). To date, analysis of epistatic interaction associated with some agronomic compositions has been extensively performed in rice (Yu et al., 1997), wheat (Singh et al., 2013), soybean (Lü et al., 2011), rapeseed (Li et al., 2012; Wang et al., 2015) as well as other corps. The study on epistatic effect facilitates us to understand if some QTL or genes hold the additive or epistatic effect in some specific environments, involving the case that some genes neutralize or suppress the expression of other genes. In this study, a total of 69 pairs of epistatic interaction loci were detected across three macroenvironments type (winter, semi-winter and spring). From the results we noted that the epistatic interaction pairs for the three FA compositions, C16:0, C18:3, and C20:1, all originated from the A8 and C3 linkage groups. In addition, except for one epistatic interaction pair arised from the interaction of C3 × C3 for C18:1, two epistatic interaction pairs from the interaction of A9 × C3 and one from the interaction of C3 × C3 for C22:1, the remaining interaction pairs were all obtained from the interaction between A8 and C3 for this two compositions. This centralized distribution pattern of these interaction pairs demonstrated that the epistatic interaction effect between A8 and C3 linkage groups played a predominant role in PV of FA concentration in *B. napus*. More investigations are needed to deepen the understanding of epistatic effect across the two linkage groups in future.

More interestingly, some special interaction pairs were repeatedly detected in multiple different microenvironments. For example, one interaction pair of EI (A8-22.8) and EI (C13-184.4) were detected in five field trials of 10DL, 11DL, 11WH, 12DL, and 12WH responsible for C18:1, C18:2, and C22:1, and for the composition of FAS, two differently distributed interaction loci of EI (A8-25.1) and EI (C13-183.5) were detected for twice in the microenvironment of 11HG and 12DL, respectively. Some interaction hotspot, such as EI (A8-22.8), EI (A8-25.1) and EI (A8-26.9), participated into the interaction process for 16, 10 and 9 times of the total 69 pairs of epistatic interaction loci, respectively. Additionally, some common epistatic interaction loci, such as EI(A8-22.8) and EI(C13-185.2) were also found in four different field compositions of 12DL, 12WH, 11DL, and 09DL for the compositions of C18:2, C18:3, C22:1, and FAS. These discoveries suggested that these epistatic loci were stably expressed and played an assignable role in the FA accumulation of acyl lipid metabolism process.

Apart from the observation of the epistatic interaction pairs, a total of 18 FA metabolism related genes were also revealed and were located near loci of epistatic interaction. The two epistatic interaction loci, EI (A8-25.9), containing two CI of 25.6–26.9, and 25.6–27.6 interacts with EI (C3-182.8) locates in the CI of 182.5-183.6 and two 3-ketoacyl coenzyme A synthase (*KCS*) family genes of *KCS17* and *KCS18*, were detected in this two QTL loci. Another epistatic locus EI (A8-25.9) located in the CI of 25.6-27.6 and interacted with the locus EI (C3-177.4). The locus of EI (A8-25.9) contained two *KCS* genes of *KCS17* and *KCS18* and EI (C3-177.4) contained one long chain acyl-CoA synthetase (*LACS*) family gene of LACS6 and the multifunctional protein (*MPF*) orthologous gene *MPF2*. Two gene of ATTLL1 and ECI2 included locus of EI (A8-22.8) with the CI of 19.9-25.1 interacted with the locus of EI (C3-183.5) with the CI range of 182.0-184.1, which involved the gene of *KCS18* and simultaneously and interacted with another locus of EI (C3-192.7) that contained the gene of *KCS17*. Another locus of EI (A8-22.8) located in the CI with the range of 19.9-24.1 and simultaneously interacted with two loci of EI (C5-109.9) and EI (C3-183.5) locus, the two genes *ATTLL1* and *ECI2* were also found in the region of EI (A8-22.8), *MFP2* and *AT3G07690* were revealed in the CI region of EI (C5-109.9) and *KCS18* was detected in the CI region of EI (C3-183.5) locus. The two epistatic loci interaction between EI (A8-25.6) and EI (C3-184.4) were also found and two genes of *KCS17* and *KCS18* were revealed in these two regions. The last gene involving epistatic interaction pair of EI (A8-23.6) and EI (A3-112.4) was found and two gene pairs of *KCS17, KCS18*, and *AT4G16155, HSD3* were discovered in each region.

It is noteworthy that despite *KCS17* and *KCS18* participated in the most epistatic interaction with other gene (Table S3), while in the analysis result of network interaction *KCS18* just directly interacted with one epistatic gene of *HSD3* only, and *KCS17*, similar with *ATTLL1*, actually did not participate into the network interaction (Figure 4). Among the 18 epistatic candidates, two pairs only existed on direct interaction in the network, except for the interaction pair of *KCS18* and *HSD3*, the other were the pair of *ECI2* (*IBR10*) and *MFP2*. These results indicated that the weak epistatic interaction effect might exist among this epistasis participated genes for the FA accumulation during the seed development. Collectively, the result of epistasis interaction demonstrated not only the single genes and/or loci underlying the QTL but also the epistatic interactions among them that played essential roles in the processes of FA accumulation and metabolism of rapeseed.

### Interaction analysis and the potential metabolic regulatory network provide more deeply knowledge for understanding FA metabolism in *B. napus*

Here, more than 13 thousand orthologous candidates of *Arabidopsis* underlying the CI of the unique QTL were revealed. To further study the genetic mechanism of FA metabolism, 61 acyl lipid metabolism related that were elicited and a complex and interrelated network was constructed. The interaction network showed that some candidates predominantly interacted with others. For example, the two most upstream and main node genes *LACS8* and *LACS9* in plastidial FA pathway closely interacted with two genes of *AIM1* and *MFP2*, both of them were the main candidates within β-Oxidation process. Additionally, 15 plastidial FA were involved and 8 of β-Oxidation involved genes were also processed in the close interaction with each other, respectively. The two processes of TAG synthesis and β-Oxidation contained the close relationship, mainly mediated by the gene of *SDP1*, which belonged to TAG degradation pathway. Intriguingly, except for the candidate of *KCS18*, the remaining eight genes within FA elongation process were relatively independent from other pathway related genes (Figure 4).

In this study, a pyruvate initiated metabolic pathway of FA was constructed, which gave a legible and intuitional direction for the exploration of the genetic mechanism of FA metabolism in *B. napus*. 47 out of the 61 FA related genes were positioned in the pathway. Generally, marker density and genome coverage level of the genetic map affect the sensitivity of QTL detection (Asíns, 2002) and eventually determine the accuracy of identification of candidate genes. To the best of our knowledge, the majority of the 61 candidates (41 out of 61) were localized for the first time in *B. napus* of our study (Smooker et al., 2011; Wang et al., 2015; Qu et al., 2017). Fifteen plastid FA biosynthesis involved in orthologous were revealed, seven of which were new localization, containing *CAC1, ACP4, LIP1, LTA2, SDRD, LACS8*, and *AT4G16155* and were wholly located in seven linkage groups of A3, A6, A9, A10, C2, C3, and C7. Ten potential candidates were detected related to the FA elongation. In addition to the three pleiotropic genes of *KCS17, KCS18* and *KCR1*, the other seven genes were all new revelation, relevant to six *KCS* family genes of *KCS*1/3/4/9/19/20 and a 3-hydroxyacyl-CoA dehydratase of *PAS2* and overall distributed into four linkage groups of A6, A9, C2 and C8. 26 potential candidates were detected here related to the TAG synthesis and degradation, except for the five genes of *OLE1, GPDHC1, LPAT5, MAGL13* and *AT5G18640*, the remaining were the novel localization through our QTL mapping analysis compared to the previous studies (Smooker et al., 2011; Wang et al., 2015; Qu et al., 2017). Furthermore, four FA β-Oxidation involved genes were also detected: the gene *AIM1* locating into ucqLA-A6-1, gene *DCI1* into ucqALA-A9-*5, MFP2* into two QTL of ucqFAS-C3-1 and ucqFAS-C5-2 with negative additive effect, and the gene *ECI2* into three QTL of ucqALA-A8-2, ucqOA-A8-2 and ucqLA-A8-3*. Two* genes, *PCAT4* and *TGD2* involved in the lipid trafficking process and were not been found in previous QTL mapping analysis of rapeseed, were detected in our study and were located into the consensus QTL of ucqFAS-C2-7, ucqALA-C2-4, ucqFAS-C2-3, ucqALA-C2-3, and ucqALA-C5-5 (Table S5).

Although diverse structural genes that functioned in the complex and hierarchical FA metabolic process were revealed, some regulatory factors, such as *WRI1, LEC1, LEC2*, also played essential role in the metabolic pathway. For example, *WRI1* controlled various genes involving the FA biosynthesis and simultaneously acted downstream of *LEC2* (Baud et al., 2007, 2010), it also activated another regulator of PII to modulate the FA composition in seeds of *Arabidopsis thaliana* (Baud et al., 2010). The two transcriptional activators *LEC1* and *LEC2* served as the key regulator in the FA biosynthesis process with multiple functional roles. Through interaction with the other regulators of *FUS3* and *ABI3, LEC1* affected seed maturation (Kagaya et al., 2005; To et al., 2006) as well as extensively affected the seed storage, oil production content in seeds and embryo development (Lotan et al., 1998; Mu et al., 2008; Tan et al., 2011). Similar with *LEC1, LEC2* was also correlated to the lipid accumulation and seed maturation (Kim et al., 2015). Surprisingly, these important regulators mentioned above were revealed in CI of the detected QTL in our study. This may result from the possibility that the PV of FA composition in our DH population might not be preferably caused by any transcription factors but the genes directly related to lipid metabolism.

In summary, numerous key genes involve in acyl lipid metabolism were discovered underlying the CI of the QTL detected in our DH population and provided clues for FA metabolism pattern in *B. napus*. Although the more complicated FA and oil biosynthesis in rapeseed than in *Arabidopsis*, with the in-depth research, especially the study of the marker-assisted gene mapping, the genetic basis of FA biosynthesis process will become more and more clear.

## Conclusion

In the present study, we implemented FA compositions related QTL mapping and identified and analyzed candidate genes based on a high density linkage map in *B. napus*. A lot of stable, environmental specific as well as many novel QTL were detected. Unconditional and conditional QTL analysis revealed numerous genetic variations and demonstrated the complicated genetic basis of FA metabolism. Through comparative genomic analysis, dozens of acyl lipid metabolism related potential candidates were detected and were positioned to a finely constructed FA metabolism pathway and interaction network of *B. napus*. Taken together, this study gave a more profound understanding for genetic basis of acyl-lipid metabolism in *B. napus* and provided a valuable guidance for breeding program of rapeseed in future.

## Author contributions

BB and HC carried out the data analysis and wrote the manuscript. HW and WZ participated in the field experiment. LZ, NR, XW and BW made helpful suggestions to the manuscript. HJ and ML designed, led and coordinated the overall study.

### Conflict of interest statement

The authors declare that the research was conducted in the absence of any commercial or financial relationships that could be construed as a potential conflict of interest.

## Supplementary material

Figure S1 Distribution frequency of the three types QTL (identified, consensus and unique) within the entire genome of *B. napus* in KN DH population. The histogram shows the distribution frequency of QTL that located in each linkage groups and the table exhibits the concrete number of the QTL for each linkage groups.

Figure S2 Overlapping region of the identified QTL hotspots for the eight FA traits on chromosome A8 and C3. The original identified QTL for different traits are showed by colored curves with respective likelihood value above the line of the linkage group (top). The colored curves from the bottom shows the additive effect value for the corresponding QTL above. The threshold equates to the likelihood value of 3.2. Different color indicates different traits and the different form of these lines indicates the different breeding environments.

Table S1 Pearson correlation analysis result for FA trait pairs in the KN DH population. The symbol “–” represents the missed correlation coefficient attributed to the simultaneously measured concentration data for the two traits. The symbol “^**^” represents the significance level at *p* < 0.01.

Table S2 The revealed identified QTL for each FA composition in KN DH population of this study.

Table S3 The epistatic QTL interaction pairs detected for each FA composition. EI: epistatic interaction, AA, additive × additive interaction type; SE, standard error; NO, no candidate genes were detected in epistatic region.

Table S4 The total revealed potential candidates underlying the CI of unique QTL of KN DH population.

Table S5 The screened FA metabolism related candidate genes underlying the CI of unique QTL. The candidate genes with bold indicated that they were located in the major QTL region. The involved consensus QTL in bold were the QTL with positive additive effect and the rest with the negative additive effect.

Table S6 The total identified and consensus QTL for the trait of oil content in KN DH population.

Table S7 The detected conditional identified QTL for each FA composition in KN DH population. The PA|SA indicates that the trait of SA conditioned on the trait of SA for the QTL analysis, the others are the same meaning.

Table S8 The detected conditional consensus QTL for each FA composition in KN DH population. The writing format of PA|SA indicates that the trait of SA conditioned on the trait of SA for the QTL analysis, the others are the same meaning.

Table S9 The primer pairs of the five candidates for the experiment of qPCR analysis.

## Abbreviations

ATHSD3: *Arabidopsis thaliana* hydroxysteroid dehydrogenase 3
OLE1: Oleosin 1
ATGRP20: *Arabidopsis thaliana* glycine-rich protein 20
CLO4: Caleosin 4
PKL: Protein kinase like 1
SL1/2: HSI2-like 1/2
GPDHC1: Glycerol-3-phosphate dehydrogenase, NAD-dependent, C-terminal 1
ASIL1: 6B-interacting protein 1-like 1
CEK1/2: Choline/Ethanolamine Kinase1/ 2
FATB: Fatty acyl-ACP thioesterases B
ACP4: Acyl carrier protein 4
LIP1/2: Lipase1/2
CAC1/2: Chloroplastic acetyl coenzyme carbxylase 1/2
LTA2/PLE2: Plastid E2 subunit of pyruvate decarboxylase 2
FAB1/2: Fatty acid biosynthesis1/2
SDRD: Short-chain dehydrogenase/reductase isoform D
EMB3147: Embryo defective 3147
KCR1: Ketoacyl reductase 1
ATTLL1: *Arabidopsis thaliana* triacylglycerol lipase-like 1
SDP1: Sugar-dependent 1
DALL1/2: DAD1-like lipase 1/2
AIM1: Abnormal inflorescence meristem 1
ECI2: Enoyl-CoA hydratase/isomerase 2
DCI1: Dienoyl-CoA isomerase 1
TGD2: Trigalactosyldiacylglycerol 2
PTAC4: Plastid transcriptionally active 4
ABI3: Aba insensitive 3
LEC1/2: Leafy cotyledon1
WRI1: WRINKLED 1
AG3P (LPA): Lysophosphatidic acid
AG3PE: Diacylglycerol 3-phosphate ethanolamine
CDP-DAG: CDP- diacylglycerol
CoA: Coenzyme A
DAG: Diacylglycerol
DAG3P (PA): Phasphatidic acid
DHAP: Dihydroxyacetonephosphate
FFA: Free fatty acid
G3P: Glycerol-3-phosphate
MAG: Monoacylglycerol
PC: Phosphatidylcholine
PE: Phosphatidylethanolamine
PGP: Phosphatidyglycerophosphate
PI: Phosphoinositides
PS: Phosphatidylserine
TAG: Triacylglycerol
QTL: Quantitative trait loci
FA: Fatty acid
DH: Doubled haploid
CI: Confidence interval
PV: Phenotypic variation
PA: palmitic acid
SA: stearic acid
OA: oleic acid
LA: linoleic acid
ALA: linolenic acid
EIA: eicosenoic acid
EA: erocic acid
OC: Oil content.
