# Supplementary figures and images for: Stable, Environmental Specific and Novel QTL Identification as Well as Genetic Dissection of Fatty Acid Metabolism in Brassica napus
Source: Front Plant Sci. 2018 Jul 17;9:1018. doi: 10.3389/fpls.2018.01018 (PMC6057442; doi:10.3389/fpls.2018.01018)

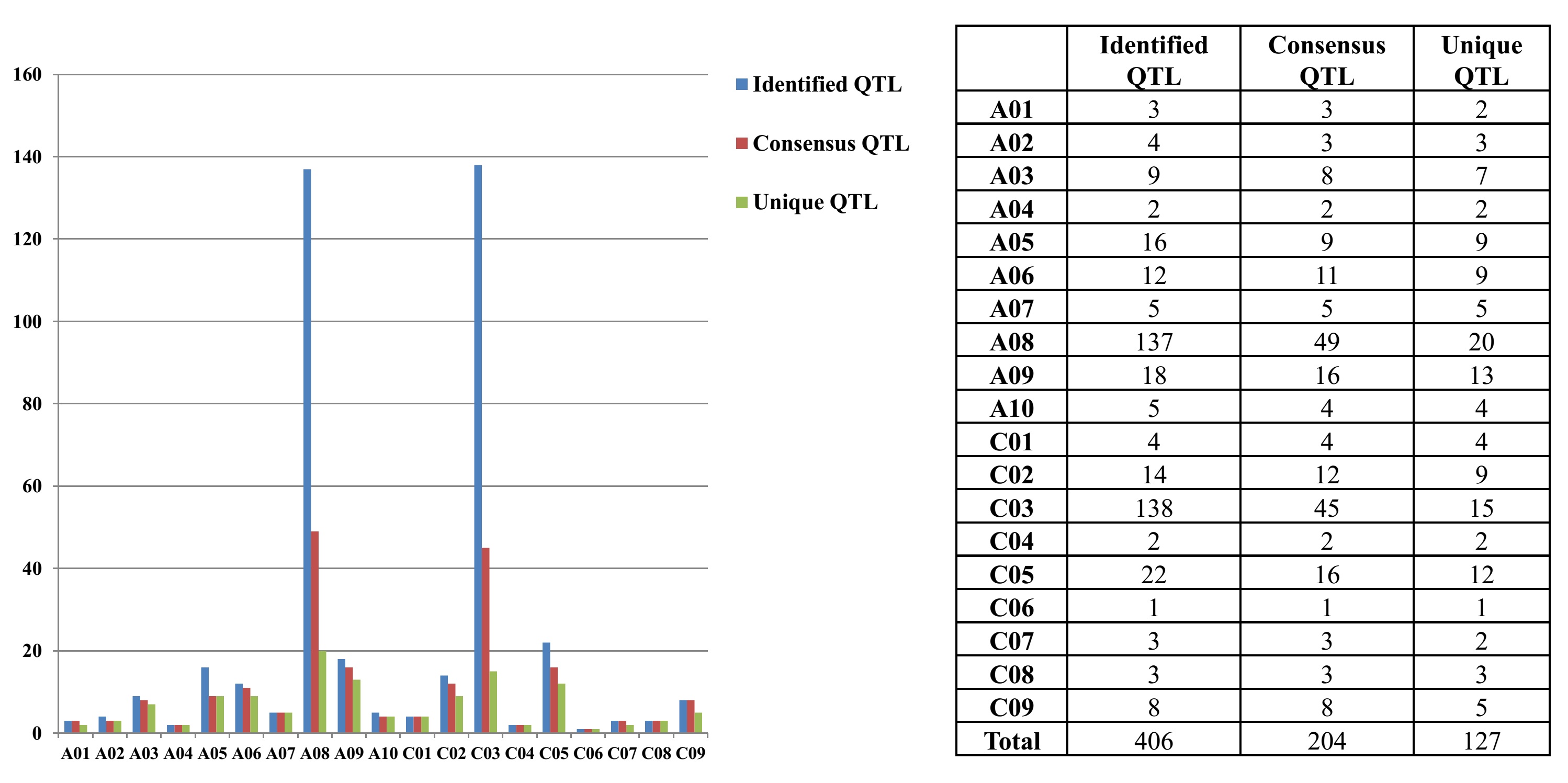

Supplement: Figure S1 — Distribution frequency of the three types QTL (identified, consensus and unique) within the entire genome of B. napus in KN DH population. The histogram shows the distribution frequency of QTL that located in each linkage groups and the table exhibits the concrete number of the QTL for each linkage groups. [file Image_1.JPEG]
